# Dynamic filopodial forces induce accumulation, damage, and plastic remodeling of 3D extracellular matrices

**DOI:** 10.1371/journal.pcbi.1006684

**Published:** 2019-04-08

**Authors:** Andrea Malandrino, Xavier Trepat, Roger D. Kamm, Michael Mak

**Affiliations:** 1 Department of Mechanical Engineering, Massachusetts Institute of Technology, Cambridge, Massachusetts, United States of America; 2 Institute for Bioengineering of Catalonia, Barcelona, Spain; 3 Unitat de Biofísica i Bioenginyeria, Facultat de Medicina, Universitat de Barcelona, Barcelona, Spain; 4 Institució Catalana de Recerca i Estudis Avançats (ICREA), Barcelona, Spain; 5 Centro de Investigación Biomédica en Red en Bioingeniería, Biomateriales y Nanomedicina, Madrid, Spain; 6 Department of Biological Engineering, Massachusetts Institute of Technology, Cambridge, Massachusetts, United States of America; 7 Yale University, Biomedical Engineering Department, New Haven, Connecticut, United States of America; University of Pennsylvania, UNITED STATES

## Abstract

The mechanical properties of the extracellular matrix (ECM)–a complex, 3D, fibrillar scaffold of cells in physiological environments–modulate cell behavior and can drive tissue morphogenesis, regeneration, and disease progression. For simplicity, it is often convenient to assume these properties to be time-invariant. In living systems, however, cells dynamically remodel the ECM and create time-dependent local microenvironments. Here, we show how cell-generated contractile forces produce substantial irreversible changes to the density and architecture of physiologically relevant ECMs–collagen I and fibrin–in a matter of minutes. We measure the 3D deformation profiles of the ECM surrounding cancer and endothelial cells during stages when force generation is active or inactive. We further correlate these ECM measurements to both discrete fiber simulations that incorporate fiber crosslink unbinding kinetics and continuum-scale simulations that account for viscoplastic and damage features. Our findings further confirm that plasticity, as a mechanical law to capture remodeling in these networks, is fundamentally tied to material damage via force-driven unbinding of fiber crosslinks. These results characterize in a multiscale manner the dynamic nature of the mechanical environment of physiologically mimicking cell-in-gel systems.

## Author summary

Many cells in the body are surrounded by a 3D extracellular matrix of interconnected protein fibers. The density and architecture of this protein fiber network can play important roles in controlling cell behavior. Deregulated biophysical properties of the extracellular environment are observed in diseases such as cancer. We demonstrate, through an integrated computational and experimental study, that cell-generated dynamic local forces rapidly and mechanically remodel the matrix, creating a non-homogeneous, densified region around the cell. This substantially increases extracellular matrix protein concentration in the vicinity of cells and alters matrix mechanical properties over time, creating a new microenvironment. Cells are known to respond to both biochemical and biomechanical properties of their surroundings. Our findings show that for mechanically active cells that exert dynamic forces onto the extracellular matrix, the physical properties of the surrounding environment that they sense are dynamic, and these dynamic properties should be taken into consideration in studies involving cell-matrix interactions, such as 3D traction force microscopy experiments in physiologically relevant environments.

## Introduction

The Extracellular Matrix (ECM) is a scaffolding medium that helps transmit mechanical signals among cells in cancer [1,2], fibrosis [3,4], vascular networks [5,6], and more generally, morphogenesis [7,8]. The mechanical and biochemical properties of the ECM impact cell behavior. The stiffness of the local environment and the tensional response from cells can induce invasive phenotypes in tumors [9–12], guide differentiation in stem cells [13], and alter vascular function [14]. The fibrillar nature and local architecture of the ECM can lead to directed cell migration [15], and increased density and alignment in the tumor stroma are correlated with more aggressive disease and worse prognosis in preclinical and clinical samples [16,17]. ECM remodeling through cell contractility is also potentially a fundamental factor in tissue folding and shaping during development [18]. It is not clear, however, how ECM spatio-temporal evolution in living systems is controlled by cells to promote physiological and pathological states.

Many studies have quantified the mechanical signals transmitted by ECMs, mostly assuming ideal ECM material properties. Studies usually derive the magnitude of forces exerted by cells through imaging of fluorescent markers tethered to the ECM [19,20]. Because it is difficult to back-calculate forces in heterogeneous, dynamic environments, these approaches rely on 3D biopolymers or 2D substrates with time-invariant mechanical responses. The spatiotemporal evolution of the ECM is however relevant in many mechanobiological processes [3,18,21,22]. For instance, in angiogenesis and vasculogenesis, together with chemical signaling driving formation or inhibition patterns [23], endothelial cells mechanically sense each other [24], and cooperate to form tubular shapes by remodeling the fibrous 3D ECM [5,6]. Furthermore, mechanical signals are amplified, resulting in long-range force transmission, when ECMs are fibrillar, via local alignment and force-driven anisotropy [4,25,26]. More complex descriptions of fibrous materials are taken into account in recent 3D studies of forces in biological processes [27].

ECM remodeling remains very challenging to decipher, despite its biological ubiquity. Remodeling entails dynamic molecular processes such as cell-fiber interactions, proteolytic degradation, and crosslinking sites binding and unbinding that ultimately lead to global changes in the ECM network. Additionally, cell-scale forces are sufficient to drive ECM remodeling, and remodeled ECMs can in turn modulate mechanosensing in cells, resulting in dynamic feedback. The dynamic mechanical states of cells and the ECM, especially in physiologically relevant conditions, are not well understood.

Here we investigate cell-induced remodeling of physiologically relevant 3D fibrillar ECMs, specifically fibrin and collagen. We focus on cell force-induced non-reversible remodeling of the ECM, which interestingly occurs on the time scale of minutes and drastically changes local architectures. By toggling the tensional state of the cell, we capture and distinguish both plastic and elastic strains in the ECM. We find that cell-generated mechanical forces are sufficient to accumulate ECM at the cell periphery in a dynamic process that depends on actin nucleation factors. We then perform computational simulations using a network model with discrete ECM fibers to examine quantitatively the impact of dynamic, filopodial-like cellular forces and reaction kinetics at the ECM component level on tension and concentration profiles within the ECM network. We lastly propose that constitutive damage and plastic softening at the continuum level are capable of recapitulating both experimental and fiber-level simulation findings. Collectively, these findings might have profound implications in mechanobiology, especially in the context of cell traction force studies.

## Results

### ECM mechanical remodeling experiments

**Fig 1:**
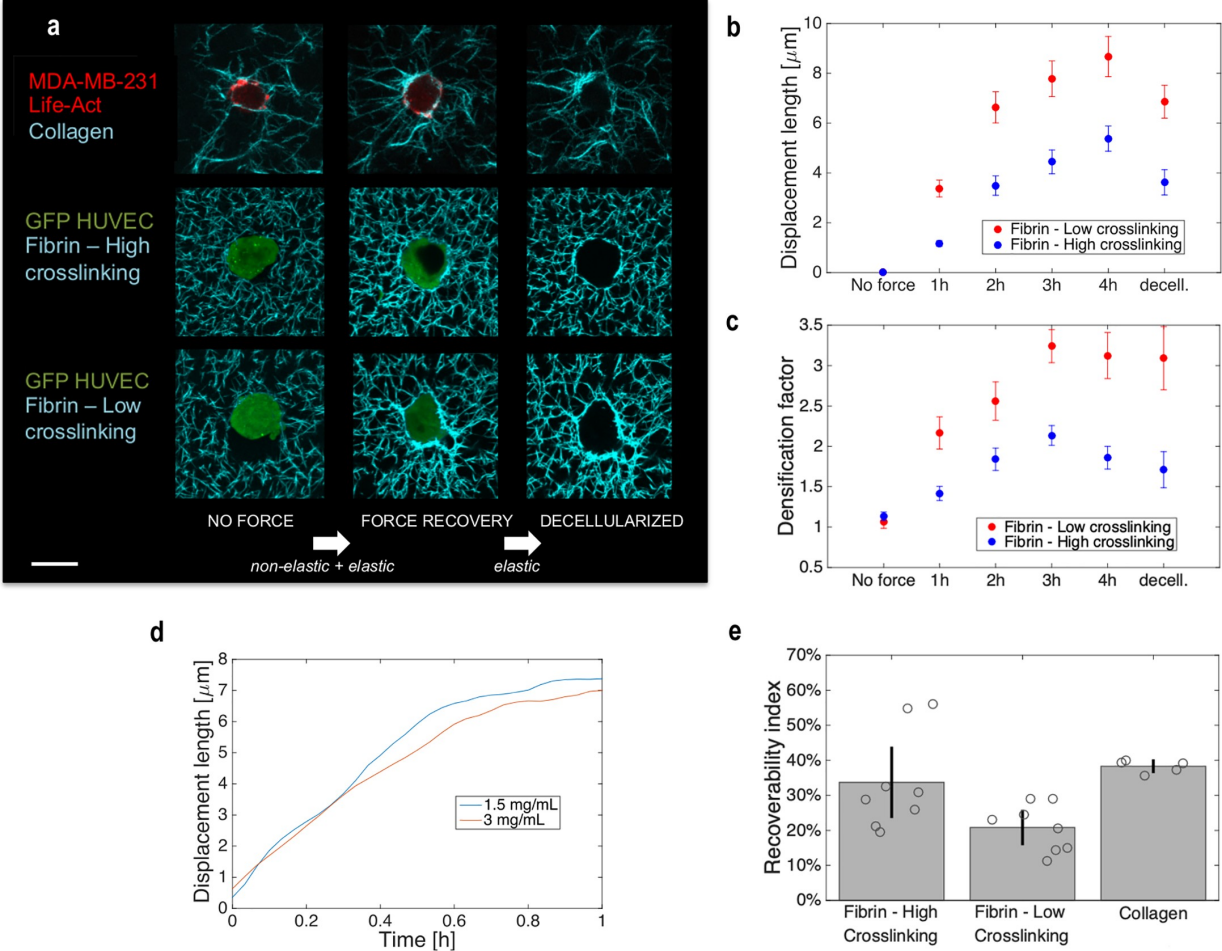
Non-elastic remodeling occurs in the course of minutes and depends on crosslinking in 3D biopolymer networks. **(a)** Z-projected confocal images of human cells including HUVECs and MDA-MB-231s suspended in 3D biopolymer networks of fibrin (3 mg/mL) and collagen (1.5 mg/mL), respectively. For fibrin gels, crosslinking was lowered using a transglutaminase inhibitor (DDITS, 0.2 mM). White arrows indicate the assumption of the type of deformation, overall (elastic *and* non-elastic) or elastic only, expected between two force configurations. Scale bar, 20 μm. (**b, c**) Quantification of the effect of crosslinking in terms of (b) displacement length (N = 8 cells per condition) and in terms of (c) densification factor (N = 7 cells per condition). Times (1h, 2h, 3h and 4 hours) are defined as the time after Cytochalasin D is removed and prior to decellularization. Decell. indicates at least 1 hour after decellularization. (**d**) Representative ECM remodeling dynamics by MDA-MB-231 cells for two different collagen gel densities. To assess the dynamics without the possible delay in force generation due to drug washout, Cytochalasin D pre-treatment was not used in these experiments. The average matrix displacement length in a ROI (~30x30 μm^2^) containing the cell and the newly recruited ECM fibers is obtained starting at the reference configuration shortly after seeding. These relatively fast dynamics measurements are calculated from time-lapse images of projected z-stacks. (**e**) Summary of *RI* for all cell-matrix pairs studied.

We explore remodeling by quantifying ECM dynamics in two physiologically relevant cell-ECM combinations cultured in 3D *in vitro* conditions. As a first combination, we use endothelial cells in fibrin gels given the well-known ability of these cells to form physiologically mimicking microvascular network structures [28]. As a second combination, we use breast cancer cells in collagen I gels, a highly abundant component in the tumor stromal microenvironment. Using both combinations, in one set of experiments, we inhibit cell-generated forces at the time of gelation and seeding by pre-treating cell-ECMs with Cytochalasin D. This treatment allows us to start from a force-free configuration (Fig 1A). After cells are seeded, we remove the Cytochalasin D and let the cells recover their ability to generate forces over a period of several hours. Finally, after recovery, we lyse the cells (decellularization) with a detergent to fully relax all forces in the cell-ECM construct. We use confocal microscopy to quantify deformations through 3D Digital Volume Correlation (DVC) algorithms using fluorescent signal intensity from pre-labeled fibrin and collagen gels [29–31].

In both cell-ECM combinations, we find that remodeling involves non-elastic, *i*.*e*. non-recoverable, deformations of the ECM. This plastic remodeling of recruited fibers is mainly force-driven; it is prevented by Cytochalasin D pre-treatment that inhibits cell-generated forces (Fig 1A). Also, increasing the crosslinking of fibrin results in a decrease in the *displacement length*, a measure of the average radial displacement of the ECM toward the cell (see Methods for details) (Fig 1B). Crosslinking has an important effect on plastic remodeling, with poor crosslinking resulting in the enhancement of fiber concentration at the cell-matrix boundary (Fig 1A–1C). For each cell, this effect is quantified by the *densification factor (DF)*, the ratio of the average ECM fluorescence intensity near the cell to that far from the cell (Fig 1C). We further quantify elastic recoverability through the metric *recoverability index* (*RI*), the ratio between the reduction in displacement length caused by decellularization and the displacement length prior to decellularization, expressed as a percent. Details of these metrics can be found in the Methods section.

For all cases, after washing Cytochalasin D from the cells, we observe a significant degree of matrix remodeling in less than 1 hour (Fig 1B and 1C). To discriminate the time scale for eliminating Cytochalasin D from the cell from that for intrinsic cell contraction, we also quantify deformations immediately after seeding without Cytochalasin D pre-treatment (Fig 1D). We demonstrate that (i) the intrinsic cell contraction and ECM remodeling dynamics occur over the course of minutes and (ii) the remodeling rate diminishes within an hour (Fig 1D, S1 Fig, and S1 Video (MDA-MB-231 cell in 3mg/mL collagen), S2 Video (MDA-MB-231 cells in 1.5mg/mL collagen), and S3 Video (MDA-MB-231 cells in 1.5mg/mL collagen, overlay of fluorescent F-actin and collagen)). All tested cell-ECM combinations exhibit a plateau in displacement length indicating that remodeling stabilizes in the course of hours with substantial irreversible components (Fig 1B and S2 Fig). For endothelial cells in fibrin, lower crosslinking increases the degree of plastic deformation corresponding to a lower *RI* (Fig 1E).

**Fig 2:**
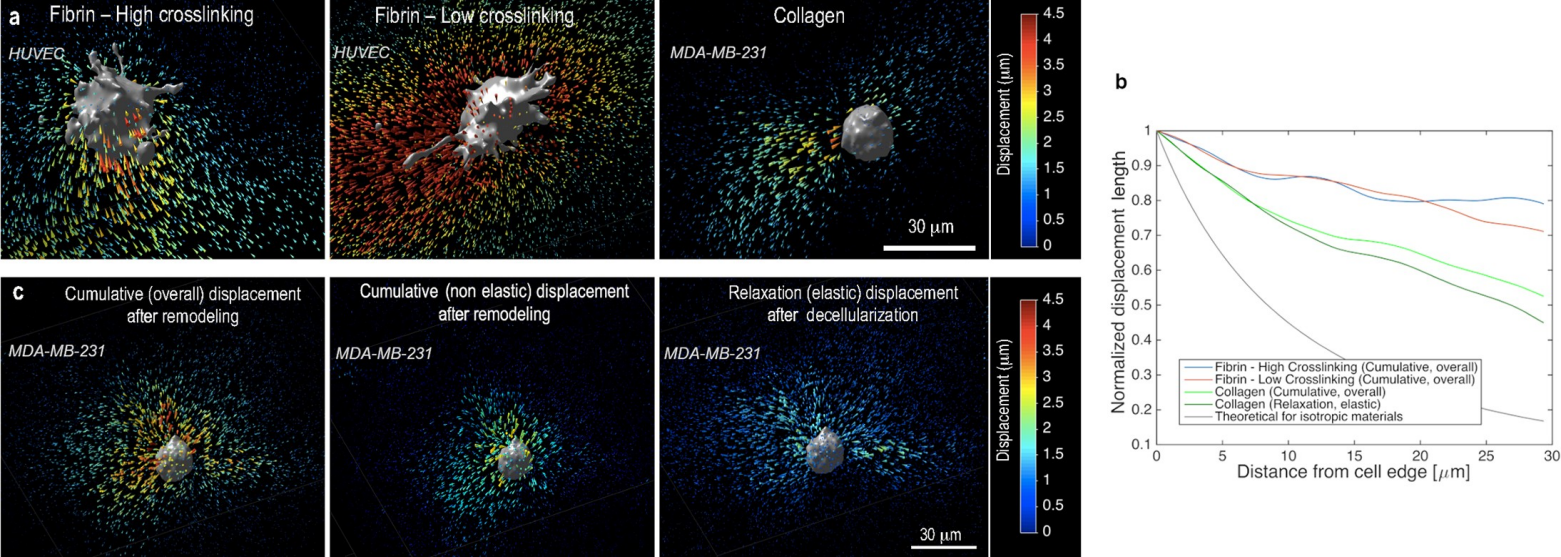
Mechanical signaling through long-range displacement propagation is cell-matrix specific and can be modified by remodeling. **(a)** 3D displacement vectors from the FIDVC algorithm, color-coded according to the magnitude of the cumulative displacement, for three representative cells in the three different matrices tested. Cumulative displacement includes both elastic and plastic components over the duration of the ECM remodeling process. (**b)** Normalized displacement length vs. distance from the cell membrane for the matrix cases analyzed (N = 5 cells per condition, with displacement lengths vs. distance being averaged from four radial directions per cell). A theoretical representation for isotropic materials, decaying as (distance)^-2^, is shown for comparison. (**c)** 3D displacement fields for a representative case of a breast cancer cell in collagen: (left) force recovery with matrix remodeling, with cumulative displacements being both plastic and elastic; (center) the plastic (non-elastic) component; and (right) force relaxation after decellularization, with displacements presumed to be the elastic component.

We further consider how remodeled ECMs absorb and transmit forces in space. In general, both endothelial and cancer cells apply centripetal tractions, as demonstrated by the directions of the local ECM displacements (Fig 2A–2C). Plastic recruitment leads to a substantially higher magnitude of cumulative matrix displacement magnitudes ‖*u*_*overall*_‖ than the elastic displacement ‖*u*_*decell*_‖ alone (Fig 2C). To assess how cell-generated ECM deformations propagate spatially, we measure the radial profile (from the cell) of ECM displacement magnitudes (or lengths), normalized by the displacement magnitude at the cell boundary (Fig 2B). We find that force transmission depends strongly on the specific cell-ECM pair, with the displacements decaying steeper in collagen than fibrin. In all cases, displacements decay with a lower gradient than in the ideal case of an isotropic linear elastic material, which confirms the long-range mechanical reach of cell forces in fibrous ECMs (Fig 2B). Fully and partially crosslinked fibrin matrices behave similarly in their ability to propagate displacements (Fig 2B, solid lines). We also observe similarities when comparing the decay of the overall displacement during remodeling to the decay of the purely elastic component of the displacement (based on measurements right before and after decellularization).

**Fig 3:**
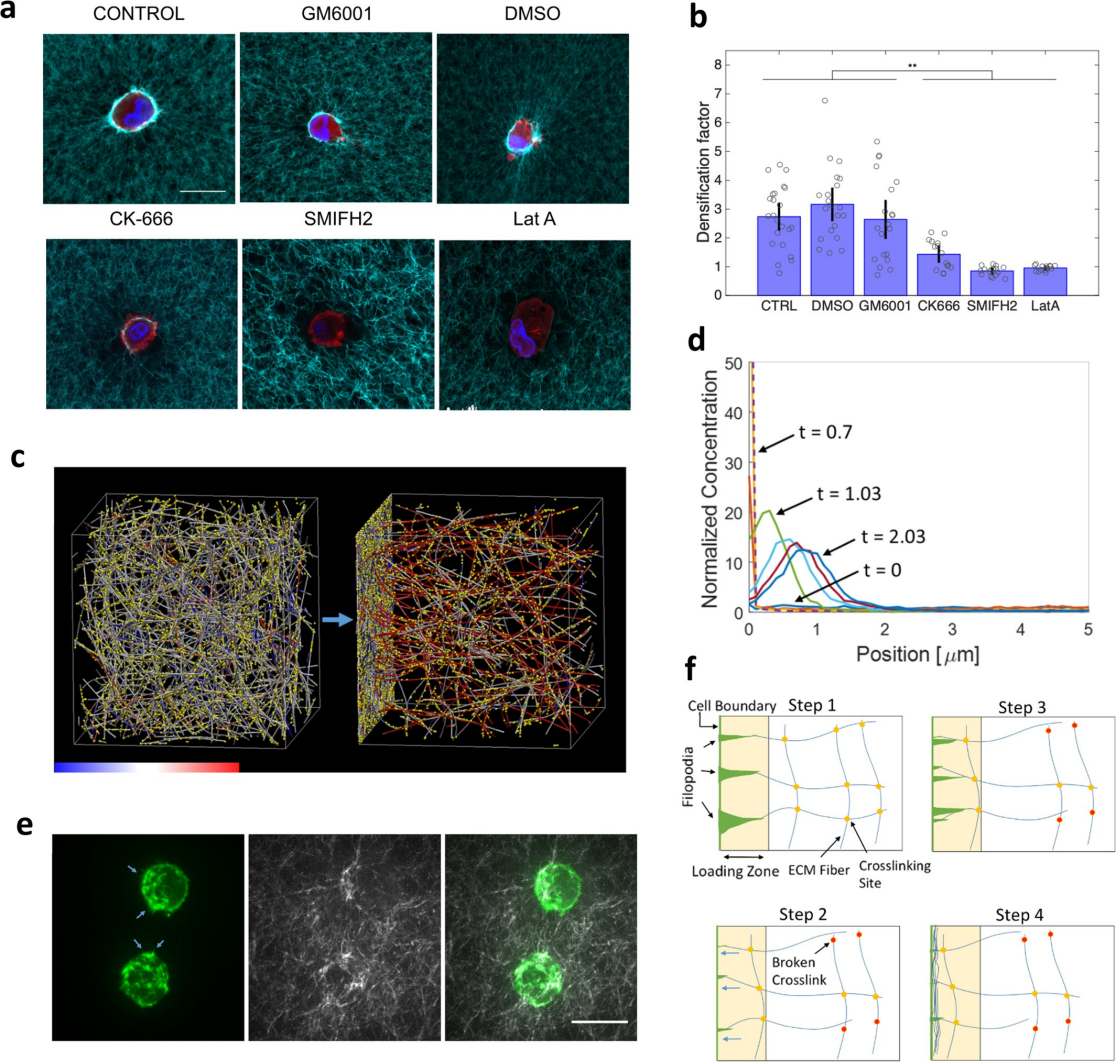
ECM recruitment by dynamic actin-driven processes. (**a**) Representative confocal images of HUVECs suspended in 3D biopolymer networks of fibrin (3 mg/mL) fixed and stained after 4h treatment with several cytoskeletal drugs and inhibitors. Blue is DAPI, red is phalloidin, and cyan is the fluorescently labeled fibrin fibers. Scale bar is 20 μm. (**b)** Statistical comparison for all treatments with drugs, in terms of densification factor (N>15 cells per case; ** p<0.01with one-way ANOVA with post-hoc Tukey HSD Test). **(c**) Computational simulation of an ECM fiber network shows network morphology before (left) and after (right) the application of loading forces near the left boundary. Colors on fibers indicate tension level according to the color bar (-300 to 300pN). Yellow spots are crosslinks (places where fiber-fiber crosslinking can occur). See also S4 Video and S5 Video. (**d)** Overlay of the time evolution of fiber concentration profiles, normalized by the initial concentration, in the force-loading direction as loading forces are exerted from the left boundary. Loading forces mimic dynamic filopodia pulling from the loading boundary, such that fibers within 2μm of that boundary experience a force pulling them toward the boundary. As new fibers or fiber segments move within that distance, new loading forces are exerted on them. Different colored curves represent different normalized times of: 0 (lower blue, uniform), 0.03 (red), 0.37 (yellow), 0.7 (purple, dashed), 1.03 (green), 1.37 (light blue), 1.7 (magenta), 2.03 (blue), where time is normalized to the total time of force application (starting at right after 0 and ending at 1). Some relaxation occurs after applied forces end, but matrix remodeling here is not reversed. The simulation setup is 100pN loading per fiber segment in the loading region, 1x crosslink zero-force unbinding rate, 0.3x crosslink mechanosensitivity, and 1x crosslink density (see S1 Table for default values). (**e)** MDA-MB-231 cells expressing fluorescent F-actin (green, left) inside a 3D collagen matrix (white, middle) with a concentration of 1.5mg/mL display many dynamic actin protrusions (blue arrows). Overlay image of actin and collagen is on the right. Images are maximum intensity z-stack projections. The scale bar is 20μm. See also S3 Video. (**f)** Schematic of ECM recruitment by dynamic filopodia. Step 1: Filopodia attach to fibers in the vicinity of the cell (loading zone). Step 2: Filopodia contract, via actomyosin-based contractile forces, pulling attached fibers toward the cell and breaking force-sensitive crosslinks. Step 3: New filopodia form and attach to new fiber regions in the loading zone. Step 4: Contraction cycle repeats, further pulling ECM fibers toward the cell. This dynamic filopodial force loading condition is applied in our discrete network simulations.

We next investigate possible mechanisms of cell force-driven remodeling at the ECM fiber scale by targeting actin nucleating factors that are important in filopodial dynamics; CK666 inhibits Arp2/3 and SMIFH2 inhibits formins. Both of these are observed to significantly reduce ECM remodeling (Fig 3A and 3B). We further show that proteolytic activity, when inhibited with GM6001 during the early remodeling process, does not appear to have a substantial effect on matrix recruitment on the timescale of hours (Fig 3A and 3B). These findings implicate dynamic cell force generation transmitted to the ECM fibers via filopodial projections in ECM accumulation, with little or no reliance on matrix degradation.

### Fiber network simulations with active remodeling

Our experimental results demonstrate that mechanical forces, mediated by dynamic actin nucleation-driven processes, and ECM crosslinking can modulate the plastic recruitment of ECM to the vicinity of cells. Here, through discrete fiber network computational simulations, we examine how the interplay between applied dynamic mechanical forces, mimicking filopodia-driven events, and kinetic fiber-fiber connections (crosslinks) lead to varying degrees of ECM remodeling and stress profiles. We extract quantitative details of how local molecular features can influence global reorganization dynamics of the ECM under cell-generated forces. A 3D fiber network, mimicking the ECM, is generated by polymerizing monomeric units into elastic fibers that can stretch and bend. Each fiber is a chain of cylindrical segments, and each segment follows the following potentials:

$$U_{s}=\frac{1}{2}\kappa_{e}\Delta r^{2}$$ (1)

$$U_{b}=\frac{1}{2}\kappa_{b}\Delta \theta^{2}$$ (2)

where *U*_*s*_ is the potential energy from stretching, *U*_*b*_ is the potential energy from bending, *κ*_*e*_ is the extensional stiffness, *κ*_*b*_ is the bending stiffness, Δ*r* is the deviation from the equilibrium length, and Δ*θ* is the deviation from the equilibrium angle. Each monomeric unit adds a cylindrical segment to the fiber during polymerization, and fibers nucleate in random directions during initial network formation. Neighboring fibers are connected with crosslinks, if present, that can unbind in a force-sensitive manner in accordance with Bell’s model [32]:

$$k_{u}=k_{u0}e^{\frac{\lambda F}{k_{B}T}}$$ (3)

where *k*_*u*_ is the crosslink unbinding rate, *k*_*u*0_ is the zero-force unbinding rate, *λ* is the mechanosensitivity (i.e. mechanical compliance) of the crosslink, *F* is the magnitude of the extensional force acting on the crosslink (only positive stretching forces contribute), *k*_*B*_ is the Boltzmann constant, and *T* is temperature. Model parameters are listed in S1 Table.

The network is athermal and the components (fiber segments, crosslinks) follow the equation of motion:

$$F_{c,i}+F_{i}-\zeta_{i}\frac{dr_{i}}{dt}=0$$ (4)

where *i* is the index of the component under consideration, ***F***_*c*,*i*_ is the cell generated loading force near the–z boundary, ***F***_*i*_ is the mechanical force from the fiber network, which includes extension, bending, and repulsion (volume exclusion) [33] of the fibers and crosslinks, *ζ*_*i*_ is the drag coefficient, and ***r***_*i*_ is the position. Eq 4 is solved over time through Euler integration at discrete time steps to determine the position of each element in the network. Crosslink unbinding is modeled stochastically. Each bound crosslink has an unbinding probability at each time step *Δt* equal to:

$$P_{unbind}=1-\exp\left(-k_{u}\Delta t\right)$$ (5)

Additional details of the discrete fiber network model, which has been applied previously to simulate other filamentous networks, can be found in [33,34]. Sample simulation are shown in Fig 3C and 3D, S4 Video (high loading forces, high ECM recruitment), and S5 Video (moderate loading forces, moderate fiber recruitment). Force loading in these simulations mimics filopodia and is described in more detail later.

Parameter values for the simulated fibers and network are chosen based on plausible values for ECM fibers (collagen I and fibrin) [35–39], and experimental network features (S3 Fig). We simulated moderately thick ECM fibers, which are ~ 100nm in diameter [40,41]. The Young’s modulus of an ECM fiber can be on the orders of tens of MPa’s for fibrin [35] and hundreds of MPa’s for collagen [37–39]. For simplicity, we picked an arbitrary value in this range (125MPa) and focused on the kinetic features of the model, driven by the force-sensitive unbinding of the crosslinks. Crosslinks have two arms, each 20nm, mimicking ECM molecular subunits that can connect fibers [42]. We explored a range of crosslink behaviors that spans relatively extreme cases (near permanent to highly transient), relative to expected fibrin bonds [36] to capture limiting network-level behaviors. For simplicity and computational feasibility, we only consider the thick fiber structures and one type of crosslink (fiber-fiber connections), which enable us to capture the dynamic connectivity of the ECM network, of focus here (S3 Fig, which shows that fiber-fiber contacts are prominent).

Once the crosslinked network is generated, it is allowed to relax to a stable state, in which the prestress built up during network formation has relaxed to a plateau close to zero. Thereafter, loading forces simulating filopodia are applied on one side of the network for a fixed duration of time and then reduced to zero to allow the network to relax to a new, potentially remodeled state. In our time series analyses, t = 0 corresponds to the initiation of force loading and t = 1 corresponds to the cessation of active forces, where t is the normalized time. In our simulations, the fiber ends at the +z boundary are fixed to mimic the resistance from fibers far away. The x and y boundaries are periodic, and the domain size is 20x20x20μm^3^. Filopodial force loading is applied such that any fiber segment that reaches within a certain distance (2μm) of the–z boundary experiences a local point force pulling it toward that boundary. We explore a range of force magnitudes from 1pN to 1nN to capture the impact of physiologically plausible cell generated forces. This loading condition mimics a pulling process where new filopodia are continuously generated that adhere to and pull new fiber segments near the cell. This type of loading is needed in order for fibers to be continuously recruited toward the cell, and many dynamic actin protrusions are indeed observed on the periphery of cells embedded inside a 3D ECM as shown in Fig 3E and S3 Video (overlay of fluorescent F-actin and collagen fibers during dynamic ECM remodeling). A schematic illustrating this loading condition is shown in Fig 3F.

**Fig 4:**
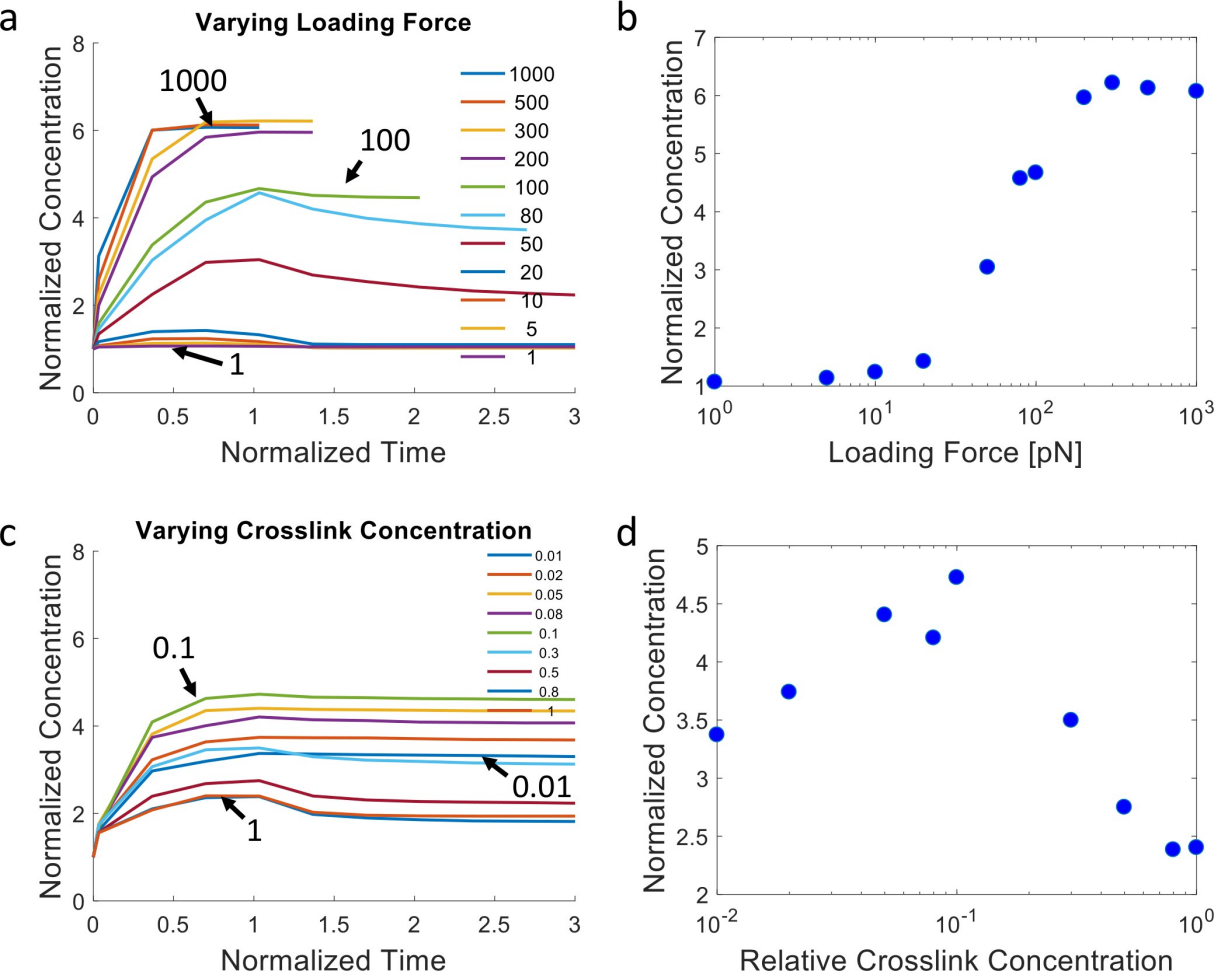
ECM recruitment profiles from discrete network simulations showing dependence on loading forces and crosslink concentration. **(a)** Normalized ECM concentration within the region 0–3μm from the cell vs. time (normalized by the duration of force application) for different loading forces (per fiber), as indicated by the arrows and color legend (in pN). (**b**) Peak normalized ECM concentration in the accumulation region as a function of the loading force. For the simulations of (a,b), the relative crosslink zero-force unbinding rate is 1x, the relative crosslink mechanosensitivity is 0.3x, and the relative crosslink density is 1x. (**c)** Normalized ECM concentration within the accumulation region vs. time for different relative crosslink concentrations, as indicated by the arrows and color legend. (**d**) Peak normalized ECM concentration in the accumulation region as a function of the relative crosslink concentration. In (c,d) the loading force is 100pN, the relative crosslink zero-force unbinding rate is 0.1x, and the relative crosslink mechanosensitivity is 0.3x. See S4 Fig for statistics of triplicate simulations for selected configurations. See S1 Table for 1x values of relative parameters.

We then examine the network remodeling dynamics under different conditions, modulating experimentally tunable or physiologically relevant parameters. Specifically, we consider different loading forces, crosslink densities, zero-force unbinding rates of crosslinks, and crosslink bond mechanosensitivities. These parameters aim to capture the impact of cell traction, the degree of ECM crosslinking, and the kinetic nature of crosslinks. ECM fibers are initially recruited to the loading boundary once applied forces are activated (Fig 3C and 3D). Temporal profiles of ECM accumulation near the cell boundary (region within 3μm of the force loading boundary) and the peak accumulated ECM concentration (over time) under varied loading forces are shown in Fig 4A and 4B, respectively. Similar plots for varied crosslink concentrations are shown in Fig 4C and 4D, respectively. The temporal profiles show that in some conditions (relatively low applied force magnitudes, high crosslink concentrations), after the loading forces are deactivated (at the normalized time of 1), the network recovers primarily elastically and the normalized ECM concentration in the accumulation region returns close to 1, the uniform network state prior to loading. Note that for high crosslinking cases, the accumulated concentration does not fully reverse after relaxation due to crosslink unbinding still having occurred, resulting in some plastic remodeling (Fig 4C). For very low loading forces, near full recovery is observed after relaxation (Fig 4A). Conversely, higher loading forces and lower crosslink concentrations lead to relatively high plastic remodeling, in which the recruited ECM fibers do not relax back to their original positions after force loading is stopped. S4 Video, S5 Video, and Fig 3D show the 3D ECM network evolution and fiber recruitment due to loading forces. In these cases, the network permanently remodels over time with recruited fibers remaining near the loading boundary after the cessation of applied forces. Note that after forces are relaxed, there are no adhesions between fibers and the force-loading boundary, mimicking decellularization in our experiments. The elastic restoring forces will then tend to pull the accumulated fibers away from the loading boundary, thus shifting the position of the maximum concentration (Fig 3D, S4 Video, S5 Video).

Furthermore, fiber recruitment vs. loading force (log scale) displays a sigmoidal trend, in which minimal ECM fiber recruitment occurs under low loading forces below a threshold, increasing ECM fiber recruitment occurs with increasing loading forces at an intermediate range, and a plateau is reached for forces above a second threshold (Fig 4B). Under the same loading forces, increases in crosslink concentration first lead to more ECM recruitment, followed by a trend reversal and decline in network remodeling (Fig 4D). This suggests that the network gains connectivity with higher crosslink concentration, enabling connected fibers farther away to be recruited. However, beyond a certain concentration, plastic recruitment is reduced, as loading forces are distributed between more crosslinks, leading to reduced crosslink unbinding rates. Note that while the crosslink reactions in our model are simulated in a stochastic manner, the overall network behavior is robust, as demonstrated by the results from repeated simulations of selected conditions (S4 Fig). We additionally explore ECM concentration profiles for different crosslink kinetics (S5 Fig). Notably, relatively high zero-force unbinding rates and mechanosensitivities, i.e. weak crosslinks, lead to plastic accumulation of the ECM, and there is a biphasic relationship between the amount of recruited ECM and the crosslink mechanosensitivity, similar to the effect of varying crosslink concentration.

**Fig 5:**
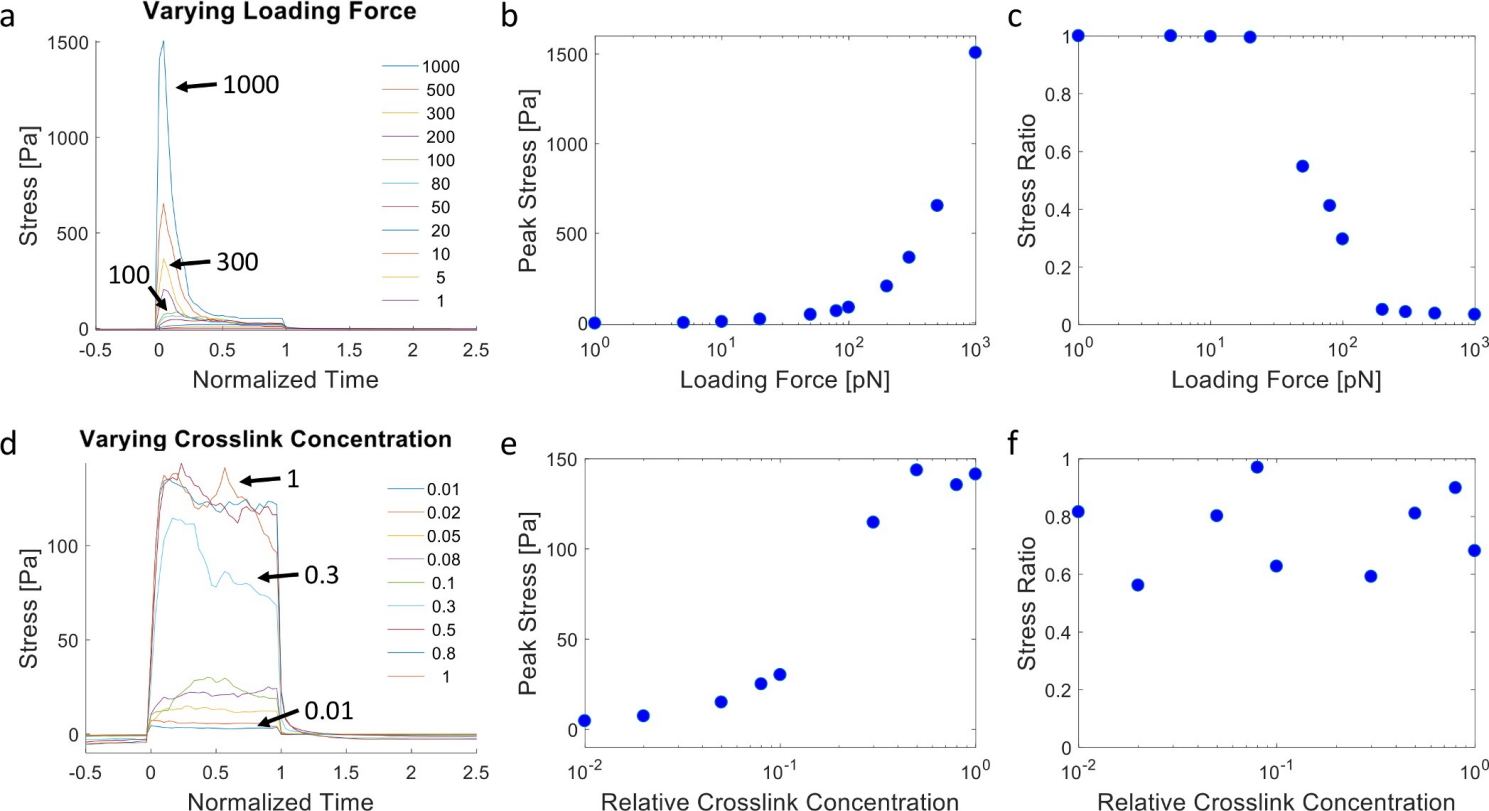
Stress generation in the dynamic ECM from discrete network simulations. **(a)** Overall stress vs. time in the ECM network during dynamic force loading for different loading force magnitudes as indicated by the arrows and color legend (in pN). (**b)** Peak stress in the network as a function of the loading force. (**c)** Ratio between the stress immediately before stopping loading forces (t = 1) and peak stress as a function of the loading force magnitude. (a,b,c) correspond to the same simulations as Fig 4A and 4B. (**d)** Overall stress vs. time in the ECM network during dynamic force loading for different relative crosslink concentrations as indicated by the arrows and color legend. (**e)** Peak stress in the network as a function of the relative crosslink concentration. (**f)** Ratio between the stress immediately before stopping loading forces (t = 1) and peak stress as a function of the relative crosslink concentration. (d,e,f) correspond to the same simulations as Fig 4C and 4D. Statistical assessment from triplicate simulations are shown in S6 Fig.

We next consider the overall stress profiles in the ECM network under our loading condition. Stresses are calculated by summing the normal component of forces acting on fibers crossing a plane parallel to the cell surface divided by the area of the plane. Stress profiles during loading (normalized time 0 to 1) are highly dynamic, often exhibiting a sharp initial peak followed by relaxation, especially under high force, as fibers are being recruited plastically and fiber crosslinks unbind (Fig 5A–5D). At relatively low applied forces, the stress profile does not decay and instead reaches a plateau, as crosslink unbinding and network relaxation are minimal during the loading period. Larger loading forces lead to larger overall peak stresses in the network, but also more unstable stresses (Fig 5B and 5C). When crosslink concentration is varied under the same loading forces, network stress is low at low crosslink concentrations as unbinding prevents a high build-up of stress. Network stress can build up to a plateaued level with more crosslink support (Fig 5D–5F, S6 Fig). Furthermore, when the kinetics of the crosslinks is tuned, more stable crosslinks lead to larger stress build-up and sustained stress levels, while crosslinks that unbind more quickly lead to reduced and less sustainable network stresses (S7 Fig). Note that the simulations discussed so far do not consider the possibility of the rebinding of unbound crosslinks. We find that enabling rebinding partially diminishes ECM recruitment and network stress dissipation (S8 Fig). Overall, our simulation results demonstrate that filopodial or filopodial-like forces acting on a kinetically connected ECM can spontaneously lead to ECM densification near the cell surface and dynamic stress profiles in the surrounding microenvironment. The amount of ECM recruitment and the temporal stress profile depend on the interplay between the magnitude of the dynamic pulling forces and the concentration and kinetic properties of the ECM crosslinks.

**Fig 6:**
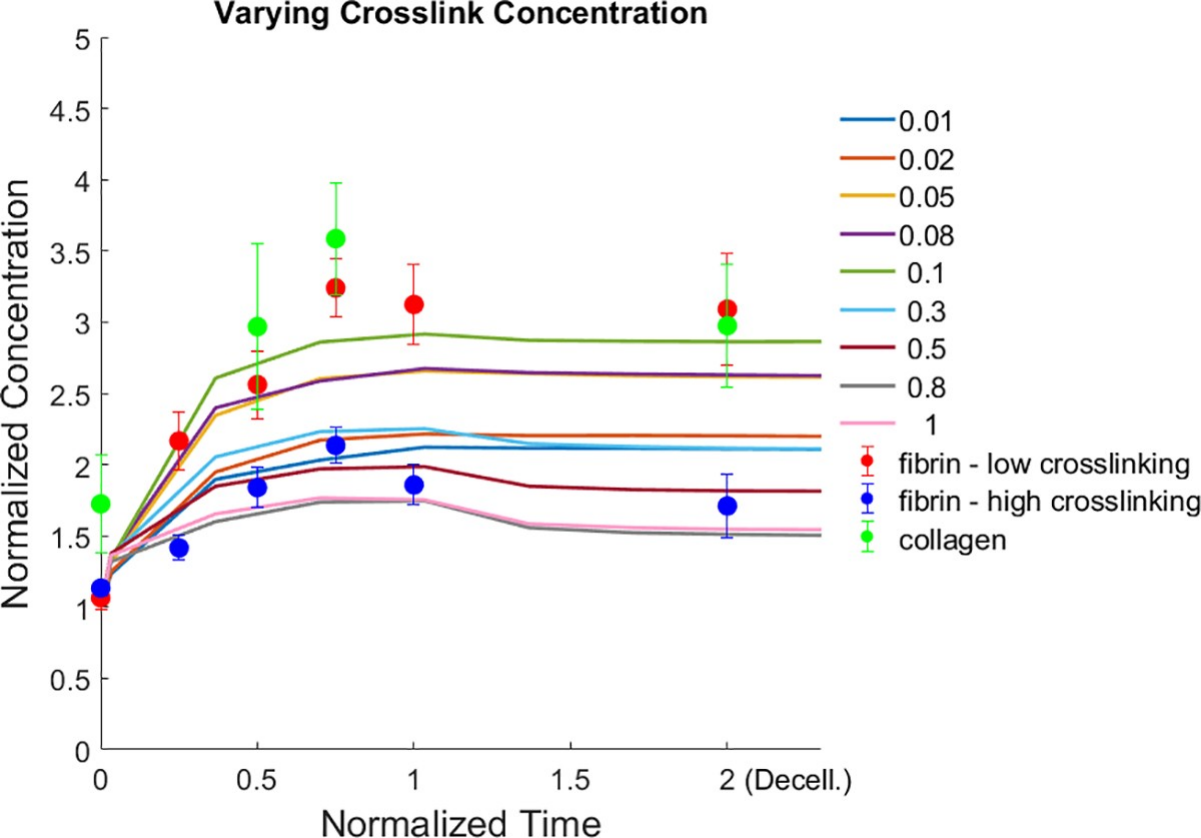
Comparison of simulations and experiments. Experimental profiles for fibrin and collagen intensities over time are compared with simulations for varying crosslink concentration. For consistency, the accumulation zone for measuring ECM density is here set to 5μm from the cell/loading surface for both experiments and simulations. For simulations, loading forces are stopped at the normalized time of 1. For experiments, time is normalized to 4 hrs, when the intensity levels appear to plateau, and experimental data points after the normalized time of 1 indicate post-decellularization as in Fig 1. Different colored curves represent simulation results with different crosslinker concentrations, as indicated in the legend. Simulation results are from the same conditions and data as in Fig 4C. Circles are experimental data, and error bars are SEM, with N = 5 cells per experimental condition.

A direct comparison of our simulation and experimental results (Fig 6) shows that by varying crosslink concentration alone in our simulations we can capture some of the differences observed in our experimental results between fibrin (low crosslinking), fibrin (high crosslinking), and collagen. Fibrin (high crosslinking) displays relatively low ECM accumulation, followed by recovery toward the initial state after relaxation, consistent with highly crosslinked simulated networks (crosslinking of 0.5–1). Both fibrin (low crosslinking) and collagen demonstrate high accumulation, much of which is non-recoverable, consistent with weakly crosslinked simulated networks (crosslinking of ~0.1).

### Continuum simulations and implication for cell traction forces

**Fig 7:**
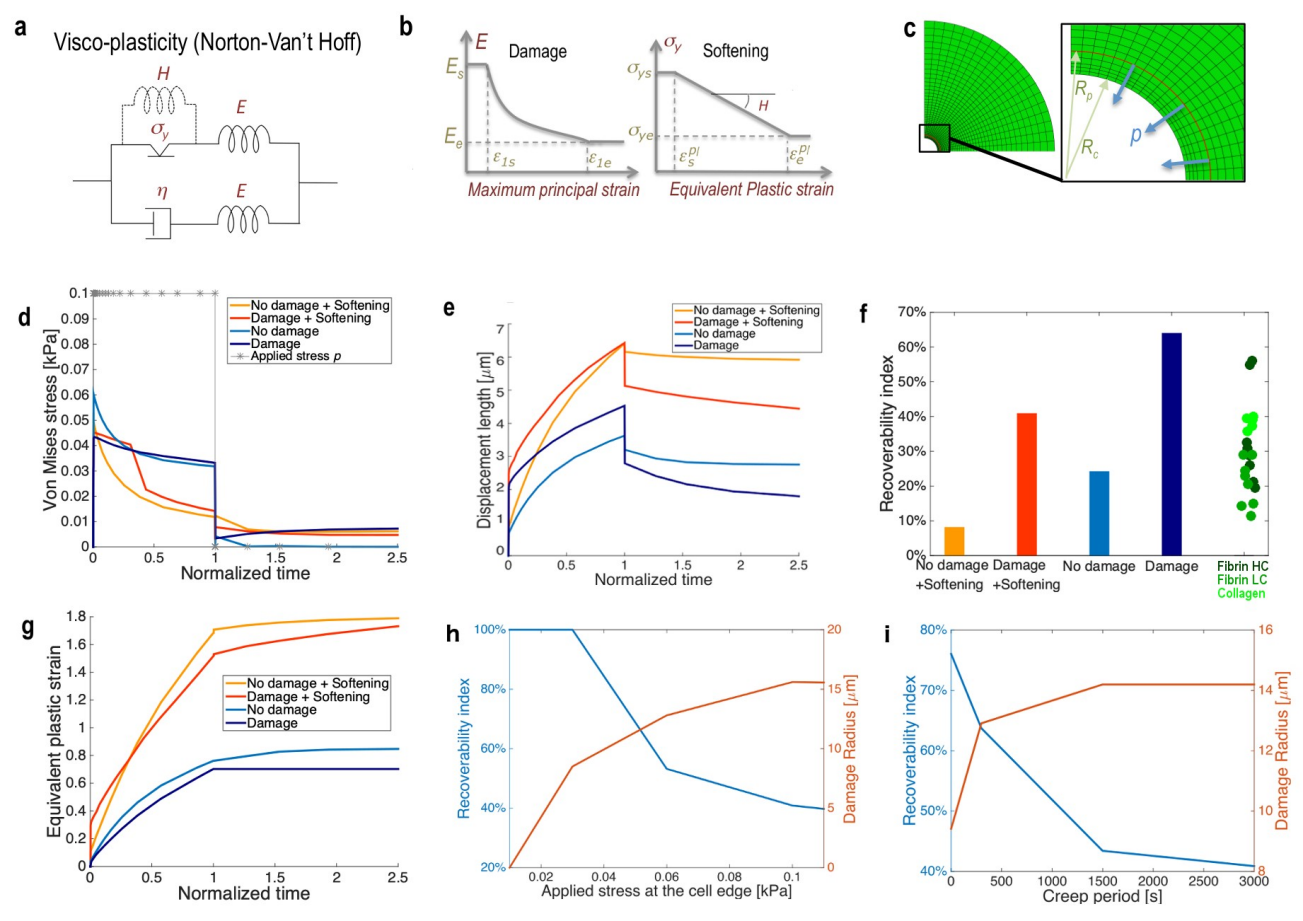
A strain-dependent plastic softening with elastic damage constitutive law recapitulates the effect of crosslinking in fibrous matrices. **(a**) Schematic of the constitutive viscoplasticity of the Norton Van’t Hoff type used to model the ECM. (Parameters provided in S2 Table). (**b**) The model is modified and alternatively tested to include phenomenologically the features of damage, simulating breakage of crosslinks with tensile strain, and plastic softening, simulating the drop in yield stress with plastic strain. (**c**) The constitutive model is implemented in the commercial finite element solver ABAQUS using existing standard viscoplastic material models and custom subroutine implementation for the damage model. An axisymmetric mesh is shown to model the ECM around a contracting cell. The zoom inset shows the surface where the load is applied to simulate the action of filopodia farther away from the edge. (**d**) Von Mises equivalent stress and (**e**) displacement length for the continuum viscoplasticity cases with or without damage and softening. Displacement lengths are calculated at the cell-ECM interface, and Von Mises stresses are calculated at approximately 5μm from the loading surface (toward the ECM), which exhibits mostly tensile stress states. In (d) the boundary applied loading history is plotted, with * showing the typical time discretization for the FE analysis. Loading starts at 0 and stops at the normalized time of 1. (**f**) Recoverability index at the cell-ECM interface (elastic deformation divided by total deformation) for the cases with or without damage and softening, along with the experimental data from Fig 1E for comparison. (**g**) Plastic equivalent strain for the cases with or without damage and softening, all calculated at the cell-ECM interface. (**h)** Recoverability index and damage radius, *i*.*e*. the radius up to regions with half-max damage (as pictured in S10 Fig), as a function of the magnitude of the applied load *p*. (**i**) Recoverability index and damage radius as a function of the creep loading time during which the load is kept constant (*p* = 0.1 kPa).

Our results implicate possible consequences for traction force microscopy (TFM) in complex 3D ECMs. In TFM studies, typically a continuum material model is used for calculating forces from strains measured through imaging fluorescent markers embedded in a deformable substrate. Here, we develop a continuum model that essentially coarse-grains fiber-level mechanics and kinetics into continuum scale parameters, and we emphasize the impact of crosslink unbinding on material properties. To capture the impact of crosslink unbinding at the continuum material scale, we utilize viscoplastic and damage features that enable creep and stress relaxation responses, as guided by our fiber network simulation results. We start from a general viscoplastic model (Norton-Hoff) (Fig 7A and S1 Note). In this model, the viscous element simulates the creep response, and the plastic element simulates permanent, inelastic deformations. To further recapitulate the effects of crosslink unbinding on the elastoplastic properties, we add both (i) ‘elastic damage’, *i*.*e*. an exponential decay in elastic stiffness (*E*) starting from above a critical maximum tensile elastic strain $(\varepsilon_{1}),\;E=Ae^{-B\varepsilon_{1}}$, and (ii) ‘plastic damage’ (softening), *i*.*e*. a linear decay of yield stress starting from above a critical plastic strain (Fig 7B). For elastic damage, we use a general form of the exponential decay, with positive parameters *A* and *B* obtained from fitting based on start and end points (*ε*_1*s*_, *E*_*s*_) and (*ε*_1*e*_, *E*_*e*_), respectively, of damage principal strain and stiffness (see below for a sensitivity study on the damage start and end points). For softening, a linear decay is used in the yield stress–plastic strain space (Fig 7B and S1 Note). Conceptually, the elastic damage feature relates to the stiffness of the material becoming lower as crosslinks unbind, and the softening feature relates to the higher unbinding rate of crosslinks as fewer bound crosslinks remain due to the increased load per remaining crosslink.

We test this viscoplastic model with damage and softening in a finite element simulation with spherical symmetry of a cell contracting centripetally and displacing the surrounding ECM with similar magnitudes as in the experiments. We then relax the contractile force to simulate the experimental decellularization. This force is applied slightly outward from the edge, simulating the action of filopodia recruiting relatively close fibers (Fig 7C). The force is meant to produce mostly tensile stresses in the continuum but also some degree of local compression at the cell-ECM interface. The presence of elastic damage recapitulates (i) the decrease in equivalent stress at the edge in perfect viscoplasticity (Fig 7D, blue lines), which is further decreased by the presence of plastic softening (red lines), in agreement with the filament model when lowering the density of crosslinks (Fig 5D) and (ii) the increase in bulk displacement at the edge with long-term loading (Fig 7E) observed when lowering the density of crosslinks (Fig 1B). However, as the elastic damage lowers the elastic modulus, it still results in more elastic recoverability than the stiffer, non-damaged material, and thus contradicting the experimental *RI* (Fig 1E). Instead, plastic softening is needed to reproduce the loss in recoverability observed experimentally as the density of crosslinks decreases (Fig 7F), which occurs in conjunction to the increase in plastic strain (Fig 7G).

We next run parametric analyses to assess how sensitive the predictions of both the elastic recoverability and the accumulation of ECM damage are to the level of force and creep time (Fig 7H and 7I). We find that both an increase of cell traction forces and persistence in loading and recruitment simulated through longer creep periods can lead to a dramatic decrease in elastic recoverability, which is accompanied by an increase of damaged regions. Thus, larger forces and longer creep times will lead to more irreversible remodeling, as also suggested by the discrete model. We also study the model sensitivity to elastic damage parameters. Note that damage is programmed to initiate at an onset strain level, *ε*_*1s*_, and ends at a saturating strain level, *ε*_*1e*_ (Fig 7B). Because both of these are elastic strain constants, we chose to link *ε*_*1e*_ to the maximum elastic strain observable experimentally during relaxation (S9 Fig), and leaving the choice of *ε*_*1s*_ as arbitrary. Nonetheless, we assess how these constants can influence the damaged region at the end of the loading process (S10 Fig). As expected, an earlier onset strain (*e*.*g*. 1%) for Young’s modulus decay will produce larger damaged regions. Also, increasing the end strain will lead to a lower damage radius, since more strain is required in order to reach larger damage levels (S10 Fig). We further show that the damage radius plateaus and can be from one to three cell diameters away from the cell edge, and it expands as the loading is held constant during creep (Fig 7H and 7I, S10B and S10C Fig, S12B Fig).

For the applicability of these continuum concepts toward quantifying cellular traction forces, we finally seek to estimate the errors introduced when elastic and plastic damage phenomena are ignored. For example, tracking the cumulative ECM deformation from an initial zero-force state via imaging, on its own, does not enable the separation of elastic and plastic deformations. An ‘apparent’ stress, back-calculated based on this total deformation and assuming linear elasticity of the ECM material, would be higher than the true stress because of plastic yield. The ratio between the true stress and the apparent stress is given by the recoverability index that has been defined and studied experimentally in Fig 1. Furthermore, a typical traction force study quantifies reference cellular stresses based on relaxing substrate deformations at the experimental end time, *e*.*g*. via cell relaxation by trypsinization, lysis, or actomyosin inhibition, and assuming nominal substrate stiffness values. Our model suggests that this quantification would be inaccurate due to plastic changes to the substrate material properties. Considering only the phenomena introduced here, *i*.*e*. elastic damage and plasticity, we estimate–with the set of parameters chosen, and experimental evidence available–that this apparent stress can again be much higher than the true stress (S11 Fig, S12C Fig, up to five time higher). We find that this is largely due to the damage process locally degrading the initial elastic modulus, but the creep duration can also have a notable effect (Fig 7I, S11 Fig, S12 Fig).

## Discussion

Many questions remain regarding the mechanics and dynamics of cell-ECM interactions in complex 3D matrices. Early studies demonstrated that cells, particularly fibroblasts, can contract collagen gels in a manner in which the degree of reversibility was dependent on the duration of contractile activity [43]. Furthermore, the mechanical properties of ECMs are irreversibly altered due to remodeling by cells [44]. These findings point toward non-elastic mechanical responses in ECMs due to cell force-mediated mechanisms. In more recent studies, confocal reflectance microscopy imaging has been widely used as a label-free technique to visualize and characterize heterogeneities in collagen matrices under cellular forces [27,45], *e*.*g*. assessing strain stiffening during cell migration [46]. Recently, using this imaging method, Mohammadi *et al*. found that fibroblasts seeded on top of a thin collagen gel induce inelastic matrix deformations, which can influence mechanosensing [47]. However, this flat “2D” cell-substrate geometry is non-physiological when considering cancer cells inside the tumor stroma or endothelial cells undergoing angiogenesis or vasculogenesis–scenarios better mimicked by cells embedded inside a 3D ECM. The geometry of the microenvironment has been shown to be critical in regulating cell phenotypes [48–50]. Moreover, confocal reflectance microscopy is a non-specific, label-free technique, which produces signals that are influenced by the presence of cells and fiber orientation, thus hindering accurate quantification of local ECM concentrations [51]. In another study, Nam *et al*. provided mechanistic, molecular-level insights into the plastic remodeling of biopolymer networks and implicated the unbinding of fiber-fiber bonds in stress relaxation, although the results were not based on direct, physiologically relevant cellular interactions with the ECM [52]. Recently, in a study using instead 3D DVC algorithms on fluorescently-labeled fibrin matrices, Notbohm *et al*. characterized the remodeling process and inferred that fibroblasts plastically push and pull fibrin to form tubular, protrusion-like permanent structures [53]. Other assessments using fluorescently labeled fibers in experiments have focused on continuum properties and characterizing the viscoplasticity of the ECM in reconstituted and living microtissues [54,55]. While useful and informative, these studies do not provide full assessment of the effects of crosslink concentration and dynamic actin-driven processes on ECM accumulation mechanics and dynamics by cells. Also, while molecular-level mechanistic insights were recognized, a multiscale computational approach linking physiological cellular forces and discrete fiber networks with local force-sensitive crosslink kinetics to a coarse-grained continuum representation of the ECM has not be fully investigated.

Our study takes an integrated computational and experimental approach toward understanding mechanical remodeling in heterogeneous, inelastic physiologically relevant biopolymer ECMs–collagen I and fibrin. We established an experimental strategy to capture sequentially the 1) initial zero-stress configuration, 2) rapid remodeling steps, and 3) final, plastically remodeled, cell stress-free state of cell-ECM systems. We used this strategy to assess the importance of the degree of crosslinking in fibrous ECMs in matrix remodeling. Increasing crosslink density has been shown to produce more than a two-fold increase in shear storage modulus, and a ten-fold increase in single filament stiffness in fibrin gels [35].

By disrupting key steps in intracellular actin dynamics, we found that ECM remodeling could be prevented, suggesting an important role for cell contraction, presumably in combination with filopodial protrusion and adhesion. This leads to a conceptual model in which the cell sends out filopodia via actin polymerization that adhere to individual matrix fibers, then retract, pulling the fibers closer to the cell. These forces then disrupt bonds between or within the matrix fibers, which may potentially form again in a new configuration, leading to matrix remodeling and plastic deformation.

Our findings confirm that mechanical force propagation is enhanced in fibrous ECMs as compared to ideally isotropic matrices. This has been previously attributed to non-linear phenomena such as strain stiffening [24], or local fiber alignment by cell-generated forces–a load-driven anisotropic effect in elastic matrices [25,26]. Here, we further show that such mechanical signals can change dynamically because the matrix is plastically remodeled–crosslink unbinding leads to force relaxation. Note that in our study, it is not possible to directly compare how far a cell can sense in collagen vs. fibrin, because different cell types were seeded in different gels at different concentrations, and we cannot precisely control cell-generated forces exerted on these biopolymers. Moreover, the same cells can have different affinities for different ECMs. However, from the normalization in Fig 2B, we can speculate that differential crosslinking or remodeling would not significantly alter displacement propagation profiles, pointing to the robustness of the connectivity of these networks for the purpose of long-range force transmission and mechanosensing [25]. These results point to the need for more investigations on the possible role of force-induced ECM remodeling in cell mechano-sensing dynamics in 3D physiological environments.

While ECM networks are rich with multiscale features that can influence their behavior, in this study we focus specifically on the roles of transient crosslinks and dynamic filopodial forces on ECM accumulation at the cell periphery. Our experimental and computational results show consistency (Fig 6) and indicate that the interplay of kinetic crosslinks and dynamic loading modulates the amount of accumulation and plastic remodeling of the ECM in the vicinity of the cell. Intuitively, molecular bonds can break under mechanical forces, and various simplified models have been developed to capture the unbinding kinetics of molecular bonds under load, including slip bonds and catch bonds, which have increased and decreased unbinding rates, respectively, when under tension [56]. For simplicity, we model crosslinks as slip bonds following Bell’s law [32]. We note that many types of bonds can exist that link ECM fibers together, such as molecular knob-hole connections that link fibrin fibrils [36], hydrogen bonds, and various intermolecular and polypeptide bonds that can slip and rupture [57]. In our simplified ECM model, we reduce the system to having only one type of bond that connects fibers, and we define that bond as a crosslink. In our simulations, we explore crosslinks with both high and low unbinding rates, thus examining the impact of both highly transient and relatively permanent bonds, providing insights toward the impact of diverse bond types (S5 Fig, S7 Fig). Future, higher resolution experimental studies and higher order, multiscale computational work can help reveal the impact of finer features on global ECM remodeling dynamics. ECM fibers are typically complex, multiscale, hierarchical structures. For example, during gelation, collagen and fibrin molecules polymerize into fibrils that bundle into thicker fibers. Thicker fibers can be linked to other fibers, forming a connected network [58,59]. The bundled fibrils in a fiber can also split, leading to branching [40]. It is possible that mechanical forces can disrupt both inter-fiber and intra-fiber bonds (fibril-fibril bonds within a fiber). It is possible that intra-fiber bonds can lead to additional effects. For example, the unbinding and rebinding of fibril-fibril bonds inside a fiber can lead to intra-fiber sliding and fiber elongation [60]. If unbinding occurs at a branch point, that can lead to the further splitting of a fiber and eventually the peeling off and breakdown of the fiber into thin fibrils. In these cases, we would expect an increase in the ratio of thinner to thicker fibers in the vicinity of the cell during force loading. Furthermore, as intra-fiber bonds unbind, it may be possible in the extreme limit for the fiber to break and fail, resulting in further relaxation mechanisms in the network.

At the coarser, material continuum level, unbinding and elastic failure of crosslinks would translate in (elastic) damage and (plastic) softening, the latter simulating the drop in average material yield stress onset, as fewer crosslinks are present. We show that both might act synergistically and be necessary to model inelastic remodeling of the ECM. Plasticity at the level of collagen fibrils has been tested experimentally and has shown high variability in plastic response, spanning from hardening to softening [61]. Further experimental assessment is needed to separate the effect of elastic damage and the effect of softening at the level of ECM fibrous hydrogels. Both effects could be present as the material is remodeled and fiber networks are re-arranged, with damage assumed to act on the elastic component and softening attributed to the plastic response to yield.

We can infer from our continuum model the effect of such elasto-plastic damaging features on the values of traction forces that one can back-calculate in a 3D experiment using these cell-ECM systems. ECM viscoplasticity has already been recently targeted for accurate descriptions of tissue dynamics [54,62]. We have additionally integrated novel experiments with discrete fiber and continuum simulations to elucidate mechanistic insights toward the dynamic physical state of the ECM during cell-matrix mechanical interactions. For instance, damage parameters can ultimately be linked to the degree of crosslinking in our experiments and discrete simulations. An early damage onset and rapid modulus decay mimic weak network crosslinking and increased force-driven crosslink unbinding, while a late damage onset and more gradual modulus decay simulate strong network crosslinking. We finally relate these experiments and simulations to a continuum reasoning that is very useful for traction force microscopy studies. Although 3D fibrous biopolymer networks have been recently characterized from a material point of view for traction microscopy [27], as well as viscoelastic inverse numerical algorithms been recently introduced for more accurate force computation [63], our study is explicitly targeting inelastic behavior of such networks. We have assessed that most traction forces would be highly overestimated in the presence of elastic damage—linked to filament-level crosslink unbinding—which is thus highlighted here as an important continuum feature that requires future experimental investigations.

Overall, our results suggest that during ECM recruitment, cells do not exhibit a stable tensional state, but rather a highly dynamic one due to relaxation from crosslink unbinding. Thus, for highly motile cells that recruit matrix, including endothelial cells and metastatic cancer cells, as they migrate to and recruit fibers from new locations inside a 3D ECM, their tensional profile is dynamic rather than static. This is a starkly different picture compared to cells on 2D artificial elastic substrates, as those cells tend to exhibit relatively stable stress profiles since the substrates do not undergo remodeling [64]. Many studies have shown that substrate stiffness affects cell tension and this, in turn, affects cell behavior. However, the dynamic state of cell tension, which appears to be characteristic of cells inside a more physiologically relevant environment of a 3D ECM, has not been fully investigated. Our study shows that certain key properties–cell loading forces, ECM crosslink density, and the kinetic nature of the crosslinks–are important in regulating this dynamic tensional state in cells, which can help guide future experiments in systematically tuning these parameters to assess cell behavior.

### Conclusion

The physiological microenvironment is often composed of a complex, fibrillar ECM that exhibits non-linear, non-elastic properties. We have demonstrated that dynamic forces generated by the actomyosin machinery are capable of mechanically reassembling the local ECM, leading to substantially increased local ECM density in the course of minutes, which is not fully reversible when the cells are relaxed. Differences in ECM ligand density can alter cell signaling and overall phenotypes [65–67]. The results demonstrated here highlight the dynamics of cell-ECM interactions in a more physiological context. The local environment sensed by cells, both physically and biochemically, is highly distinct from acellular matrices and gels in their initial states, with nominal concentration values based on stock solutions. ECMs with active cells are rapidly remodeled by cells to generate heterogeneous local environments with significantly different ligand densities and architectures. This behavior is often not considered, as only nominal ECM concentrations are usually reported, and is not captured by widely used non-physiological, elastic substrates that cannot be plastically remodeled by cells. Physical properties of the microenvironment have already been shown to lead to diverse ramifications in cell behavior, from guiding stem cell differentiation to modulating tumor dissemination and tissue morphogenesis. Our results directly implicate cell mechanics–the actomyosin machinery and dynamic filopodial or filopodial-like forces–in driving active remodeling of the ECM and the creation of new microenvironments that can dynamically modulate cell behavior.

## Methods

### Cell culture

For fibrin experiments, we culture Human Umbilical Vein Endothelial Cells (HUVEC) (Lonza) on collagen I-coated flasks in EGM-2 (Lonza) growth medium and used in experiments between passages 6–8. For collagen experiments, we culture MDA-MB-231 cells expressing fluorescent actin filaments (via LifeAct, gift from the Lauffenburger Lab at MIT) were cultured at 37°C, 5% CO2 with DMEM supplemented with 10% fetal bovine serum and 1% penicillin-streptomycin.

### Gel preparation and encapsulation

Both rat tail collagen I, solubilized in 0.02N acetic acid, (Corning) and bovine fibrinogen proteins (Sigma) are fluorescently labeled in stock solutions. A fluorescent reactive dye binding to free amine groups (Alexa Fluor 647 NHS Succinimidyl Ester, ThermoFisher) is used to produce cell-compatible, purified gels that can be visualized in 3D confocal live imaging with no known alterations of functionality of monomers reported in previous mechanobiology studies of the ECM[31,53,60]. Stock solutions are purified from the unreacted dye by using dialysis cassettes (Thermo Fisher) with a 7 kDa molecular weight cut-off. Fluorescently labeled fibrin is then obtained by mixing over ice (i) bovine fibrinogen dissolved in PBS (Lonza) at twice the final concentration (6 mg/mL) and (ii) bovine Thrombin (Sigma), dissolved at 2U/mL in EGM-2 growth medium with HUVECs. Briefly, HUVEC’s are spun down at 1200 rpm for 5 min and the cell pellet is resuspended in EGM-2 growth medium containing the thrombin and mixed with the fibrinogen solution at a 1:1 ratio. The mixture is quickly pipetted into a microfluidic device using the gel filling ports. The device is placed in a humidified enclosure and allowed to polymerize at room temperature for 10 min before fresh growth medium is introduced before the experiment to hydrate the gel.

For the lowering crosslinking in fibrin gels, a synthetic inhibitor of transglutaminase (1,3-dimethyl-4,5-diphenyl-2[2(oxopropyl)thio] imidazolium trifluoromethyl-sulfonate) (DDITS, Zedira) is used. Fibrin gels were polymerized in the absence (high crosslinking) and presence (low crosslinking) of 0.2 mM DDITS. This method has been characterized previously in fibrin gels[35]. Furthermore, it has been shown at the molecular level that in the absence of this inhibitor, there is ligation of the γ chains and α chains of fibrin, which results in an increase in instantaneous bulk stiffness[68,69].

Collagen gels are prepared by mixing acid solubilized type I rat tail collagen with a neutralizing solution (100mM HEPES buffer in 2X phosphate buffered saline at pH 7.3) at a 1:1 ratio and then diluting with 1× PBS and suspended cells in media to a final collagen concentration of 1.5 mg/mL[70]. The final solution is then allowed to gel in a humidified chamber at 37°C and 5% CO_2_.

### 3D chambers

Microfluidic devices with gel and media chambers are used because of the convenient fluid flow access for on-stage media and reagent exchange necessary for the experiments. Device design and protocol are described previously[71]. Briefly, 130 μm thick devices were fabricated using PDMS soft lithography. The chambers are 1.3 mm wide and are injected with the gel encapsulating cells. Similarly shaped chambers for media flank these gel chambers and allow the quick washing and re-introduction of small volumes of reagents in all stages of the experimental procedure.

### Experiments and quantitative measurements

For assessing the effect of cytoskeletal drugs on densification and plasticity, the fluorescently labeled gels are polymerized together with cells treated with each of the tested drugs at the following concentrations: Latrunculin A (Calbiochem, 0.8 μM), GM6001 (Calbiochem, 10 μM), CK-666 (Sigma, 100 μM) and SMIFH2 (Sigma, 50 μM). These working concentrations, for substantial inhibition effects, were taken from literature data [72,73]. Control cases–both untreated and vehicle (1μL/mL dimethyl sulfoxide (DMSO), which is the maximum concentration for drug-treated conditions)–are included in the study. Cells are cultured for 4h with the same concentration of drug in the culture media, fixed with PFA4% and stained (DAPI, phalloidin). In the dynamic force recovery experiments, the gels are polymerized together with cells treated with Cytochalasin D (Santa Cruz Biotechnology, 5 μM), which is an inhibitor of actin polymerization and leads to highly diminished cellular force generation [74]. First, images are captured of cells encapsulated in the 3D ECMs under the action of Cytochalasin D to have a force-free initial configuration. Second, the chambers are washed through the microfluidic channels with fresh media on-stage three times to remove the Cytochalasin D, and to observe the onset of ECM remodeling. During this process, fluorescently labeled fibers are imaged at small time increments and these sequential images are cross-correlated through the Fast Iterative Digital Volume Correlation (FIDVC) algorithm [30] to determine the 3D displacement field while remodeling occurs. After plastic remodeling of the ECM begins to plateau (~4h), a non-ionic detergent (Triton X, 0.1%) that preserves the gel structure while permeabilizing the cell membrane is used to lyse cells. Thus, active cellular forces are fully relaxed, as cells are eliminated from the system, at the final fiber network configuration [19]. To obtain an estimation of the remodeling dynamics without the delay from Cytochalasin D recovery, FIDVC-based displacement estimations are also performed on time-lapse videos of cells right after seeding.

We apply three key metrics to quantify our experimental data: the *displacement length*, the *densification factor (DF)*, and the *recoverability index (RI)*. The *displacement length*, ‖*u*‖, is defined as the spatially averaged displacement magnitude, computed from FIDVC, inside a 60x60x60μm^3^ ROI containing one cell. *DF* is calculated as *I*_*dens*_*/I*_*far*_, where *I*_*dens*_ is the integral of the radial intensity profile calculated within 5 μm from the cell membrane (averaged over 4 profiles per cell) and *I*_*far*_ is the integral of the intensity profile of a 5 μm line far away from the cell (averaged over 4 profiles per cell). *RI* is defined as the percent from the ratio between the displacement length caused by decellularization and the displacement length right before decellularization (*i*.*e*. overall displacement length), *RI* = 100×‖*u*_*decell*_‖/‖*u*_*overall*_‖. Note that *RI* aims to quantify elastic recoverability, as decellularization is expected to cause elastic relaxation, ‖*u*_*decell*_‖~‖*u*_*elast*._‖, and the overall displacement contains both elastic and non-elastic deformations, *i*.*e*. ‖*u*_*overall*_‖~‖*u*_*elast*.+*non*−*elast*._‖.

All confocal images in Figs 1 and 2 and related quantifications were acquired with a 20× objective and an Olympus IX81 microscope (Olympus America, Inc.). Images in Fig 3 and related quantifications were acquired with a 60× or 63× oil-immersion objective using a spinning disk confocal microscope (Yokogawa) or a spectral confocal microscope (SPE, Leica microsystems).

## Supporting information

S1 Video Fast remodeling dynamics in collagen network (3mg/mL). The time step between frames is 120s, and displacement fields (both arrows and contours) are superimposed onto the corresponding confocal time lapse images (maximum intensity z-stack projections) using image transparency. The color bar indicates the cumulative displacement range in μm. The contour plot is cropped to focus on the cells and to reflect the exclusion of regions with potential boundary artifacts from the Digital Image Correlation algorithm. The cell boundary, from tracing the corresponding fluorescent (LifeAct) MDA-MB-231 cell, is depicted in black in the first and last frames.
(AVI)

S2 Video Fast remodeling dynamics in collagen network (1.5mg/mL). Image and plot details are the same as in S1 Video.
(AVI)

S3 Video Actin and fiber recruitment dynamics. MDA-MB-231 cells expressing fluorescent F-actin (green) embedded inside a 1.5mg/mL collagen gel (white) exhibit dynamic actin protrusions during ECM recruitment. The video frame rate is accelerated 1000x and the scale bar is 20μm. Images are maximum intensity z-stack projections.
(AVI)

S4 Video Simulation of ECM fiber recruitment (high). The domain size is 20x20x20μm^3^_._ See captions of Fig 3C and 3F for color and force loading schemes. The simulation setup is 100pN loading per fiber segment in the loading region, 1x crosslink zero-force unbinding rate, 0.3x crosslink mechanical compliance, and 1x crosslink density.
(AVI)

S5 Video Simulation of ECM fiber recruitment (moderate). The domain size is 20x20x20μm^3^_._ See captions of Fig 3C and 3F for color and force loading schemes. The simulation setup is 50pN loading per fiber segment in the loading region, 1x crosslink zero-force unbinding rate, 0.3x crosslink mechanical compliance, and 1x crosslink density.
(AVI)

S1 Fig Fast remodeling through fluorescent intensity local accumulation. Representative ECM intensity dynamics due to fast local remodeling by MDA-MB-231 cells in collagen gels of different concentrations. Percent change in intensity is calculated as (I_a_/I_i_-1)*100 where I_a_ is the average intensity at specific times and I_i_ is the average intensity at the beginning of the experiment. Intensities are averaged over a ROI (~30x30 μm^2^) containing the cell and the newly recruited ECM fibers.
(TIF)

S2 Fig ECM fibrin and collagen local displacement profiles in the experiment. Displacement length over time for MDA-MB-231 cancer cells in collagen matrices and for HUVECs in fibrin matrices, (i) starting from inhibition of forces, (ii) gradually recovering forces up to 4 hours (max recovery), and (iii) ending with complete decellularization that removes all cell forces applied onto the ECM.
(TIF)

S3 Fig Fluorescently labeled fibrin. 3D fibrous microenvironment rendering of a fibrin ECM network used in the experiments. Scale bar is 5 μm.
(TIF)

S4 Fig Statistical assessment of ECM concentration profiles for selected simulations. **(a,b)** Triplicate simulations for the indicated loading forces (pN), corresponding to the network configurations and plots of Fig 4A and 4B. (**c,d)** Triplicate simulations for the indicated relative crosslink concentrations, corresponding to the network configurations and plots of Fig 4C and 4D. Error bars are s.e.m.
(TIF)

S5 Fig Additional ECM profiles from discrete network simulations and statistical assessment for robustness. **(a)** Normalized ECM concentration at the accumulation region vs. time for different relative zero-force crosslink unbinding rates, as indicated by the arrows and color legend. (**b)** Peak normalized ECM concentration in the accumulation region as a function of the relative zero-force crosslink unbinding rate. For the simulations of (a,b), the loading force is 100pN, the relative crosslink density is 1x, and the relative crosslink mechanosensitivity is 0.3x. (**c,d)** Selected configurations from (a,b) are simulated 3 times each for statistical assessment. (**e)** Normalized ECM concentration at the accumulation region vs. time for different relative crosslink mechanosensitivities, as indicated by the arrows and color legend. **(f)** Peak normalized ECM concentration in the accumulation region as a function of the relative crosslink mechanosensitivity. For the simulations of (e,f), the loading force is 100pN, the relative zero-force crosslink unbinding rate is 1x, and the relative crosslink density is 1x. (**g,h)** Selected configurations from (e,f) are simulated 3 times each for statistical assessment. Error bars are s.e.m.
(TIF)

S6 Fig Statistical assessment of stress profiles for selected simulations. **(a–c)** Triplicate simulations for the indicated loading forces (pN), corresponding to the network configurations and plots of Fig 5A–5C. (**d–f)** Triplicate simulations for the indicated relative crosslink concentrations, corresponding to the network configurations and plots of Fig 5D–5F. Error bars are s.e.m.
(TIF)

S7 Fig Additional overall stress profiles from discrete network simulations. **(a)** Overall stress vs. time in the ECM network during dynamic force loading for different relative zero-force crosslinking unbinding rates as indicated by the arrows and color legend. (**b)** Peak stress in the network as a function of the relative zero-force crosslink unbinding rate. (**c)** Ratio between the stress immediately before stopping loading forces (t = 1) and peak stress as a function of the relative zero-force crosslink unbinding rate. (a–c) correspond to the same simulations as S5A and S5B Fig. (**e–g)** Selected configurations from (a–c) are simulated 3 times each for statistical assessment. (**h)** Overall stress vs. time in the ECM network during dynamic force loading for different relative crosslink mechanosensitivities as indicated by the arrows and color legend. (**i)** Peak stress in the network as a function of the relative crosslink mechanosensitivity. (**j)** The ratio between the stress immediately before stopping loading forces (t = 1) and peak stress as a function of the crosslink mechanosensitivity. (h–j) correspond to the same simulations as S5E and S5F Fig. (**k–m)** Selected configurations from (h–j) are simulated 3 times each for statistical assessment. Error bars are s.e.m.
(TIF)

S8 Fig Effects of crosslink rebinding. Temporal profiles of the normalized concentration at the loading edge (**a**) and stress (**b**) of an ECM network with a crosslink density of 1x, zero-force unbinding rate of 1x, and mechanical compliance of 0.3x. The loading force is 100pN, and force loading starts at the normalized time of 0 and ends at the normalized time of 1. Crosslink rebinding is allowed (red) or not allowed (blue).
(TIF)

S9 Fig Example of local ECM strains as computed from the experiments. (**a**) Absolute maximum principal strain (AMPS) around an MDA-MB-231 cell in collagen in a 3D sliced view. (**b**) In-plane slice view of AMPS around an MDA-MB-231 in collagen ECM for (top) 4h experiment showing cumulative overall–plastic and elastic–deformation and (bottom) showing relaxation–elastic component–after 1h treatment with cell lysant. (**c**) In-plane slice view of cumulative and relaxation AMPS for HUVEC in fibrin ECM.
(TIF)

S10 Fig Parametric study of local damage in the continuum model. **(**a**)** Maximum principal strain parameters that control the damage model exponential decay (Fig 7B, reported also on the left) are varied. The baseline (*ε*_*1s*_ = 0.02 or 2% and *ε*_*1e*_ = 0.06 or 6% strains) and three additional variations are shown in the figure along with their effect on the spatial profile of the Young’s modulus (undamaged Young’s modulus *E*_*s*_ is 1 kPa, and fully damaged Young’s modulus *E*_*e*_ is 0.2 kPa, see S2 Table). These plots are organized in two rows depending on whether the Young’s modulus damaging process is shown at the end of 300s creep (constant loading with *p* = 0.1 kPa), or 1 hour creep. On the right, a magnification shows how the damage radius is calculated, *i*.*e*. the radius up to regions with half-max damage. For clarity, the locations of the loading application and cell in the model are also indicated. (**b**) 2D heat map of damage radius as a function of the start, or onset damage strain *ε*_*1s*_ and end strain *ε*_*1e*_ at the end of 300s creep loading (*p* = 0.1 kPa) and (**c**) at the end of 1 hour creep. Note that the heat maps show reduced parameter space because the condition *ε*_*1s*_ <*ε*_*1e*_ must hold.
(TIF)

S11 Fig Local ECM stress-strain cycles of loading, creep, and unloading, and example of stress overestimation if no damage and plasticity are considered in traction force relaxation. (**a,b**) Stress-strain response in viscoplastic (red) vs. purely elastic (blue) ECM continuum models. Different loading regimes are indicated along with loading rate (a) with damage and (b) without damage included in the constitutive material formulation. Note that in the viscoplastic models, plastic softening causes a drop in yield stress as plastic deformation increases during creep (creep period = 1500s, red dashed lines). (**c,d**) Local (at the load application region) errors in stress back-calculation based on comparing the viscoplastic and the purely elastic models of (a,b). All models have the same initial elastic stiffness: dashed grey lines represent the initial stiffness of undamaged Young’s modulus of ~1 kPa. (**c**) Comparison with damage in both elastic and viscoplastic formulations and (**d**) comparison without damage. Note that as expected, error is null with a purely elastic model with no damage (d, blue line) is calculated. The small error in d (red line) is due to a competition between softening (producing a negative slope during creep) and viscous effects during unloading. Note also that the stress axis limit is highly rescaled in (c) as compared to the same profiles in (a) to help visualize the magnitude of the error, indicated by the vertical solid line.
(TIF)

S12 Fig Damage profiles and corresponding error in local stress as a function of distance from edge. (**a**) Example of the spatial profile of the damaged Young’s modulus in the continuum model of the ECM. (**b**) Radial profile of Young’s modulus in two times of the load history: upon increasing the load *p* linearly from 0 to 0.1 kPa (blue), and at the end of 1 hour creep (red). (**c**) Spatial variation of the ratio between *apparent* stress–as if no ECM damage or plasticity was considered–and *real* stress. This ratio is estimated as in S11 Fig.
(TIF)

S13 Fig Schematic of the Norton-Hoff viscoplasticity with hardening/softening capability (light colored spring). (TIF)

S1 Table Parameters used for the discrete model simulations. (DOCX)

S2 Table Parameters used for the finite element continuum model simulations. (DOCX)

S1 Note Viscoplasticity theory and finite element calculations. (DOCX)
