# Supplementary figures and images for: Dynamic filopodial forces induce accumulation, damage, and plastic remodeling of 3D extracellular matrices
Source: PLoS Comput Biol. 2019 Apr 8;15(4):e1006684. doi: 10.1371/journal.pcbi.1006684 (PMC6472805; doi:10.1371/journal.pcbi.1006684)

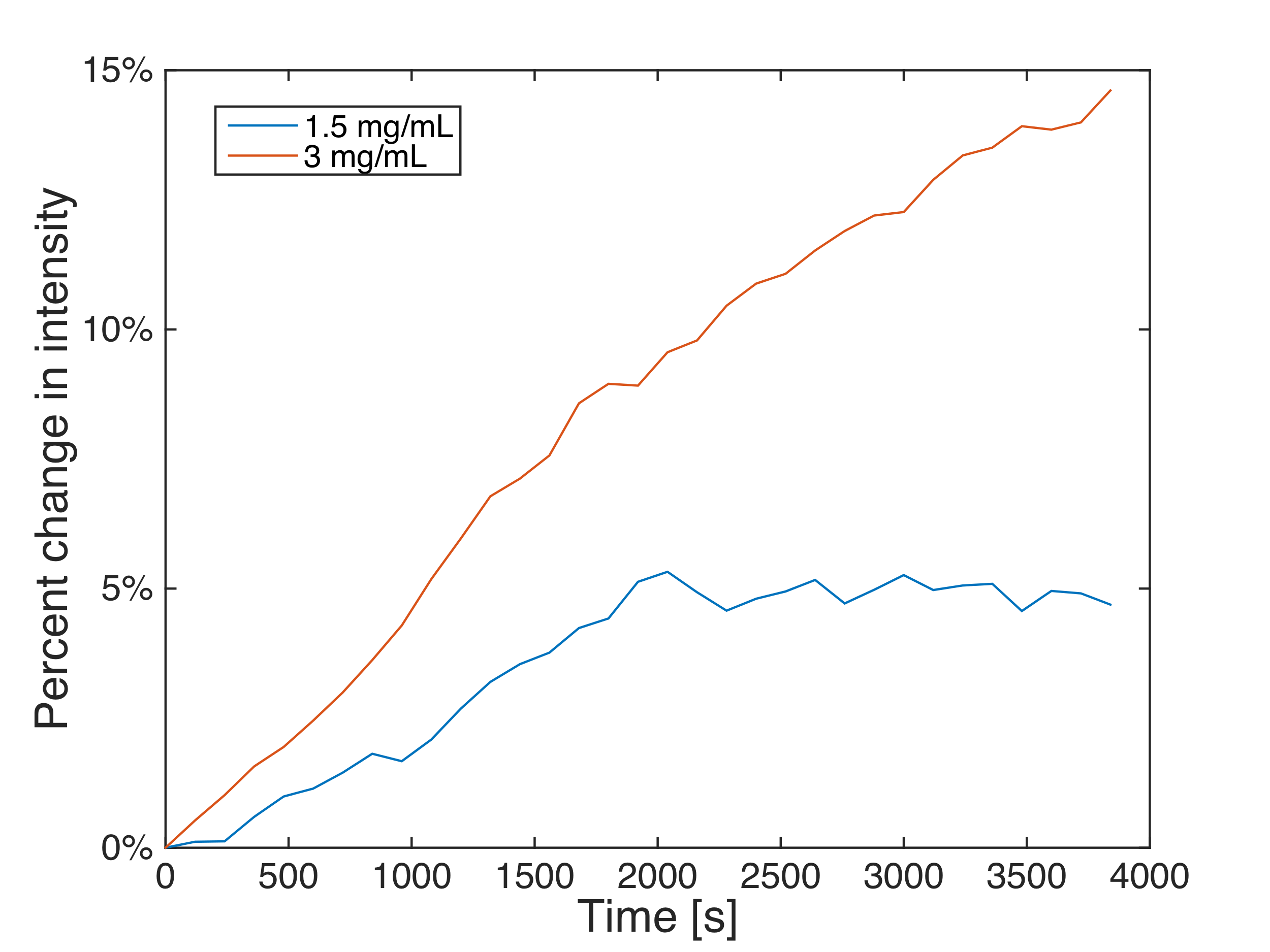

Supplement: S1 Fig — Representative ECM intensity dynamics due to fast local remodeling by MDA-MB-231 cells in collagen gels of different concentrations. Percent change in intensity is calculated as (Ia/Ii-1)*100 where Ia is the average intensity at specific times and Ii is the average intensity at the beginning of the experiment. Intensities are averaged over a ROI (~30x30 μm2) containing the cell and the newly recruited ECM fibers. (TIF) [file pcbi.1006684.s006.tif]

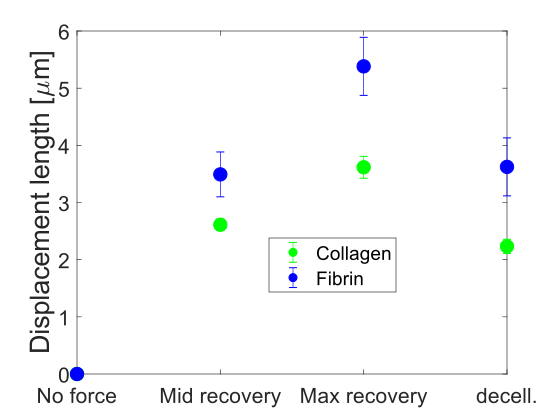

Supplement: S2 Fig — Displacement length over time for MDA-MB-231 cancer cells in collagen matrices and for HUVECs in fibrin matrices, (i) starting from inhibition of forces, (ii) gradually recovering forces up to 4 hours (max recovery), and (iii) ending with complete decellularization that removes all cell forces applied onto the ECM. (TIF) [file pcbi.1006684.s007.tif]

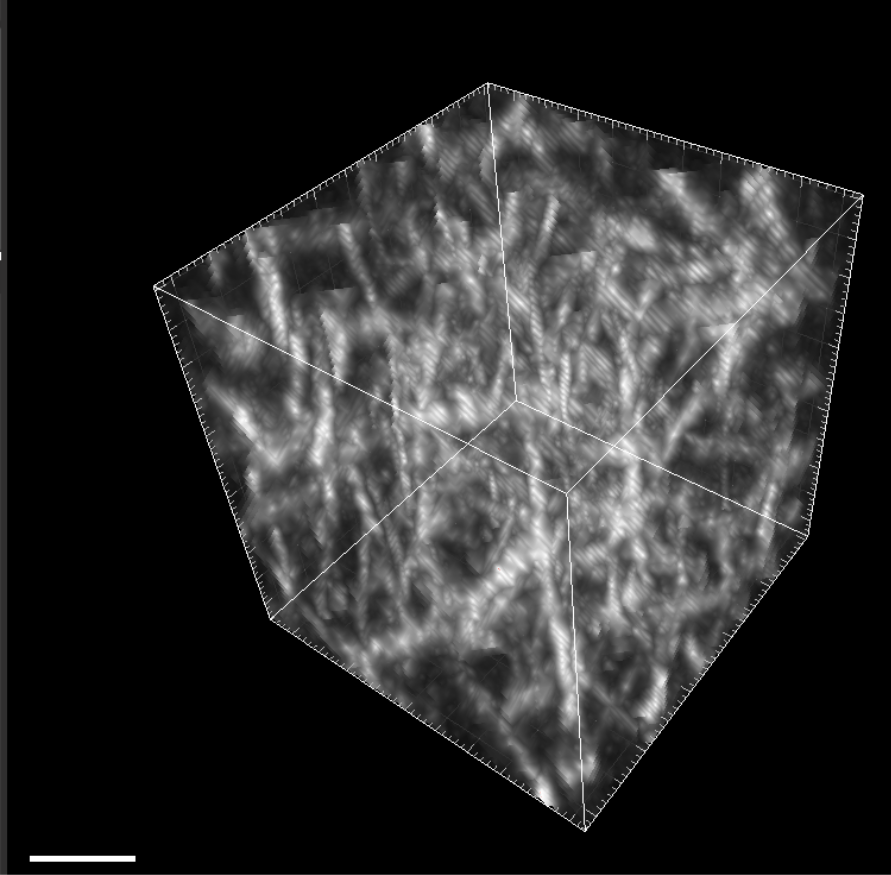

Supplement: S3 Fig — 3D fibrous microenvironment rendering of a fibrin ECM network used in the experiments. Scale bar is 5 μm. (TIF) [file pcbi.1006684.s008.tif]

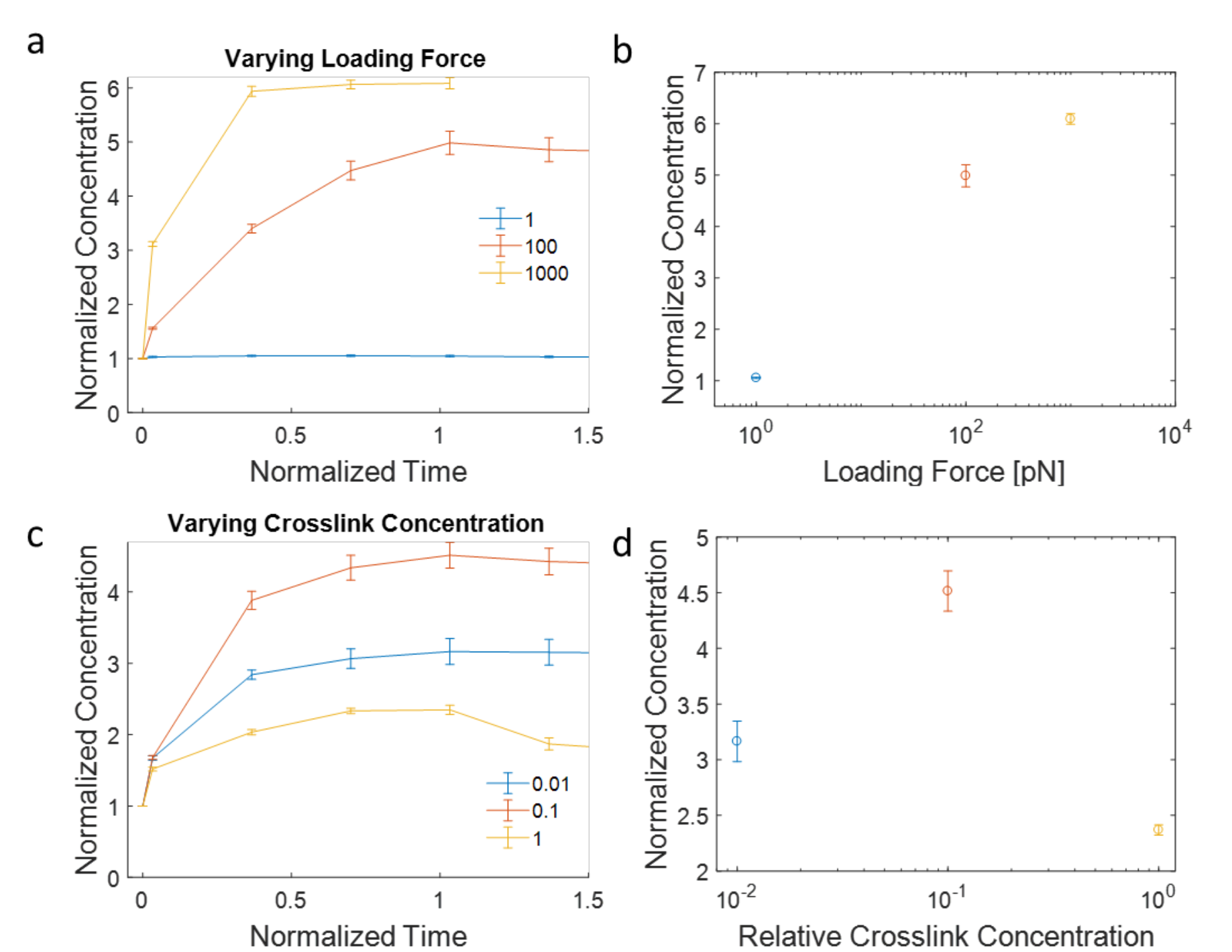

Supplement: S4 Fig — (a,b) Triplicate simulations for the indicated loading forces (pN), corresponding to the network configurations and plots of Fig 4A and 4B. (c,d) Triplicate simulations for the indicated relative crosslink concentrations, corresponding to the network configurations and plots of Fig 4C and 4D. Error bars are s.e.m. (TIF) [file pcbi.1006684.s009.tif]

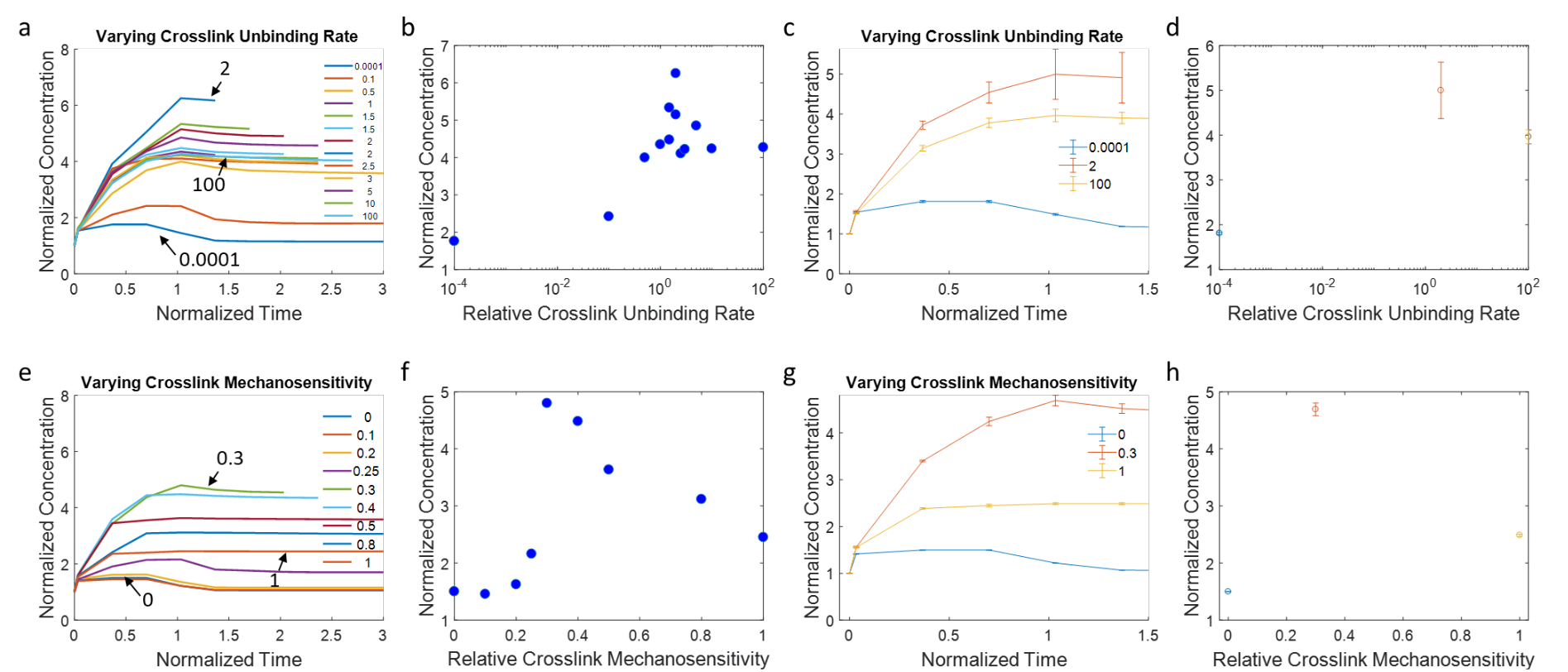

Supplement: S5 Fig — (a) Normalized ECM concentration at the accumulation region vs. time for different relative zero-force crosslink unbinding rates, as indicated by the arrows and color legend. (b) Peak normalized ECM concentration in the accumulation region as a function of the relative zero-force crosslink unbinding rate. For the simulations of (a,b), the loading force is 100pN, the relative crosslink density is 1x, and the relative crosslink mechanosensitivity is 0.3x. (c,d) Selected configurations from (a,b) are simulated 3 times each for statistical assessment. (e) Normalized ECM concentration at the accumulation region vs. time for different relative crosslink mechanosensitivities, as indicated by the arrows and color legend. (f) Peak normalized ECM concentration in the accumulation region as a function of the relative crosslink mechanosensitivity. For the simulations of (e,f), the loading force is 100pN, the relative zero-force crosslink unbinding rate is 1x, and the relative crosslink density is 1x. (g,h) Selected configurations from (e,f) are simulated 3 times each for statistical assessment. Error bars are s.e.m. (TIF) [file pcbi.1006684.s010.tif]

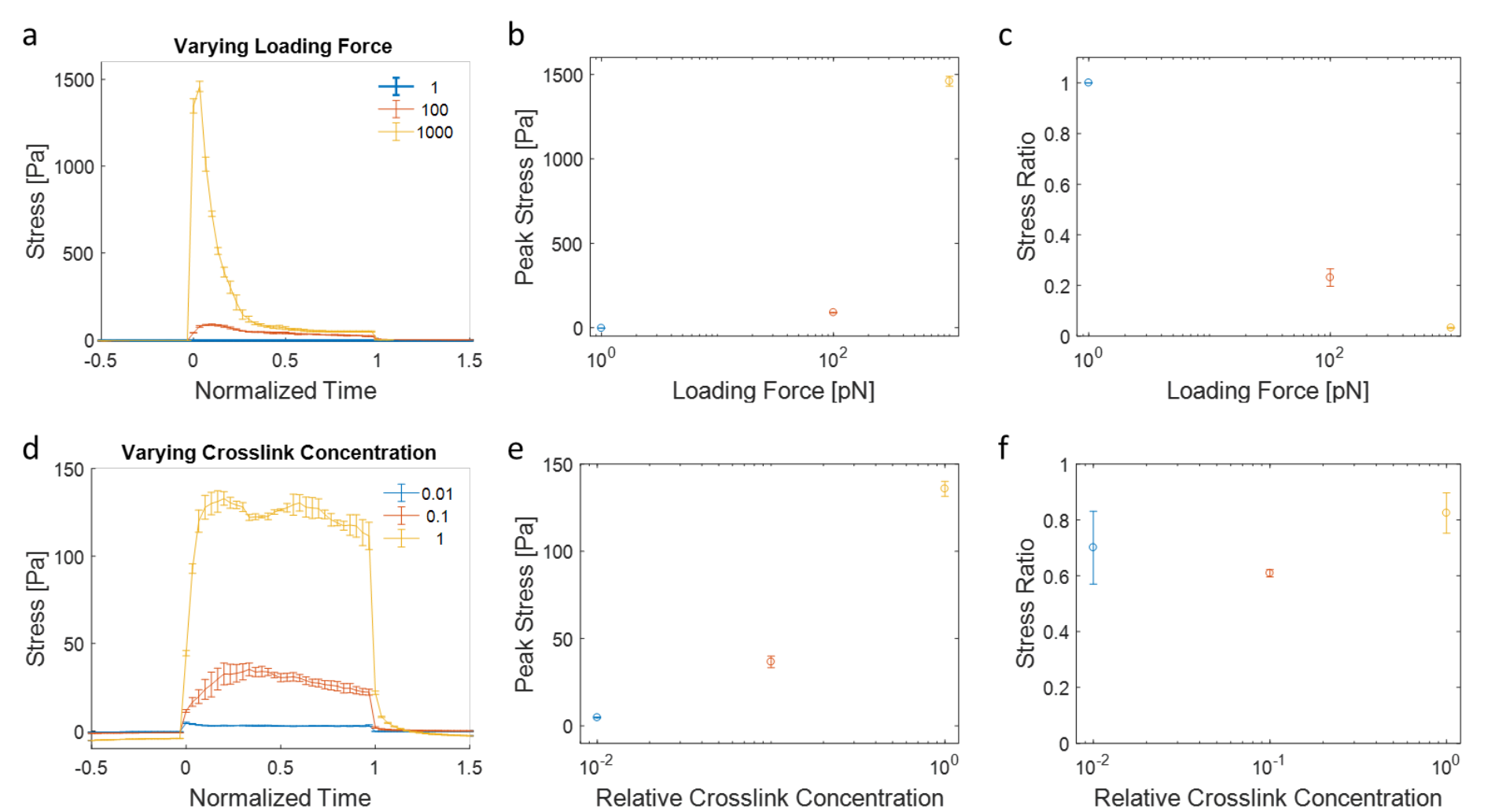

Supplement: S6 Fig — (a–c) Triplicate simulations for the indicated loading forces (pN), corresponding to the network configurations and plots of Fig 5A–5C. (d–f) Triplicate simulations for the indicated relative crosslink concentrations, corresponding to the network configurations and plots of Fig 5D–5F. Error bars are s.e.m. (TIF) [file pcbi.1006684.s011.tif]

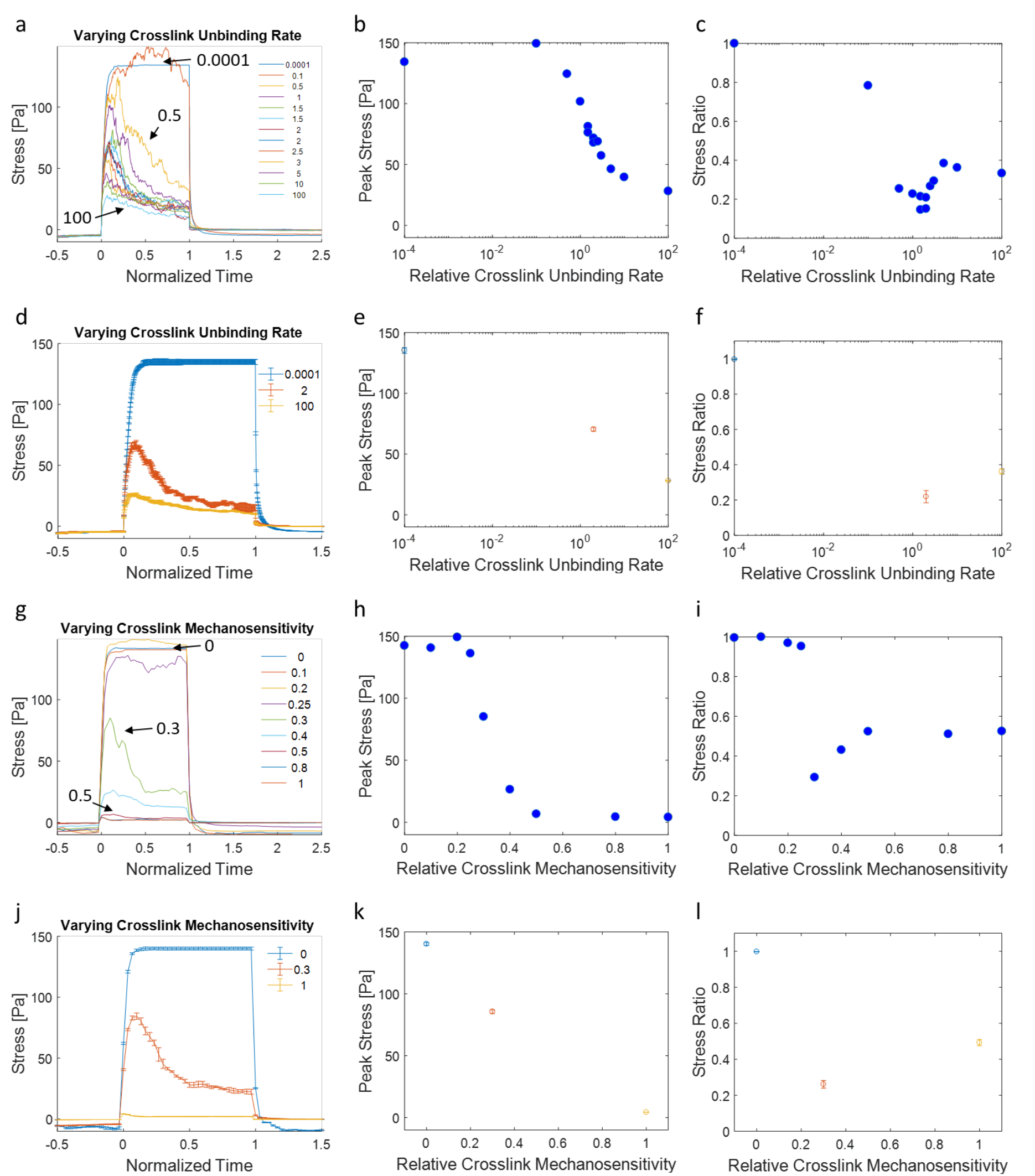

Supplement: S7 Fig — (a) Overall stress vs. time in the ECM network during dynamic force loading for different relative zero-force crosslinking unbinding rates as indicated by the arrows and color legend. (b) Peak stress in the network as a function of the relative zero-force crosslink unbinding rate. (c) Ratio between the stress immediately before stopping loading forces (t = 1) and peak stress as a function of the relative zero-force crosslink unbinding rate. (a–c) correspond to the same simulations as S5A and S5B Fig. (e–g) Selected configurations from (a–c) are simulated 3 times each for statistical assessment. (h) Overall stress vs. time in the ECM network during dynamic force loading for different relative crosslink mechanosensitivities as indicated by the arrows and color legend. (i) Peak stress in the network as a function of the relative crosslink mechanosensitivity. (j) The ratio between the stress immediately before stopping loading forces (t = 1) and peak stress as a function of the crosslink mechanosensitivity. (h–j) correspond to the same simulations as S5E and S5F Fig. (k–m) Selected configurations from (h–j) are simulated 3 times each for statistical assessment. Error bars are s.e.m. (TIF) [file pcbi.1006684.s012.tif]

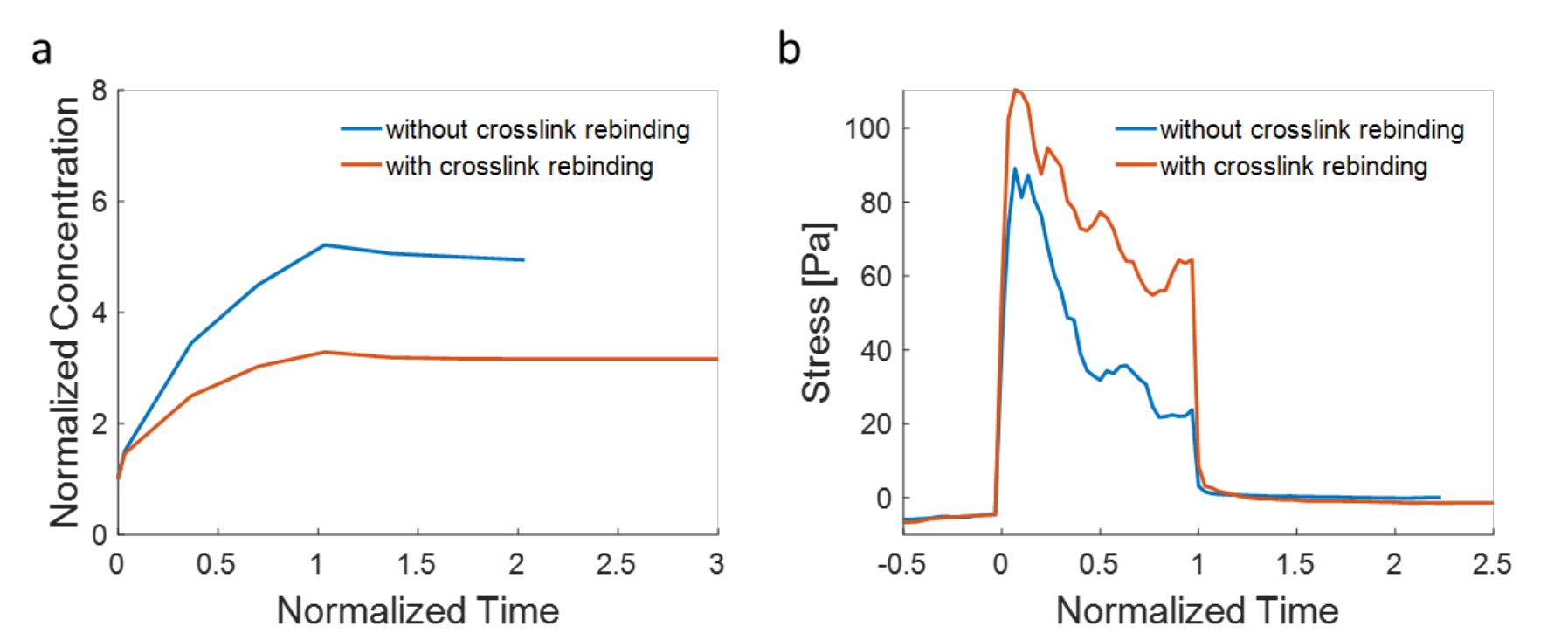

Supplement: S8 Fig — Temporal profiles of the normalized concentration at the loading edge (a) and stress (b) of an ECM network with a crosslink density of 1x, zero-force unbinding rate of 1x, and mechanical compliance of 0.3x. The loading force is 100pN, and force loading starts at the normalized time of 0 and ends at the normalized time of 1. Crosslink rebinding is allowed (red) or not allowed (blue). (TIF) [file pcbi.1006684.s013.tif]

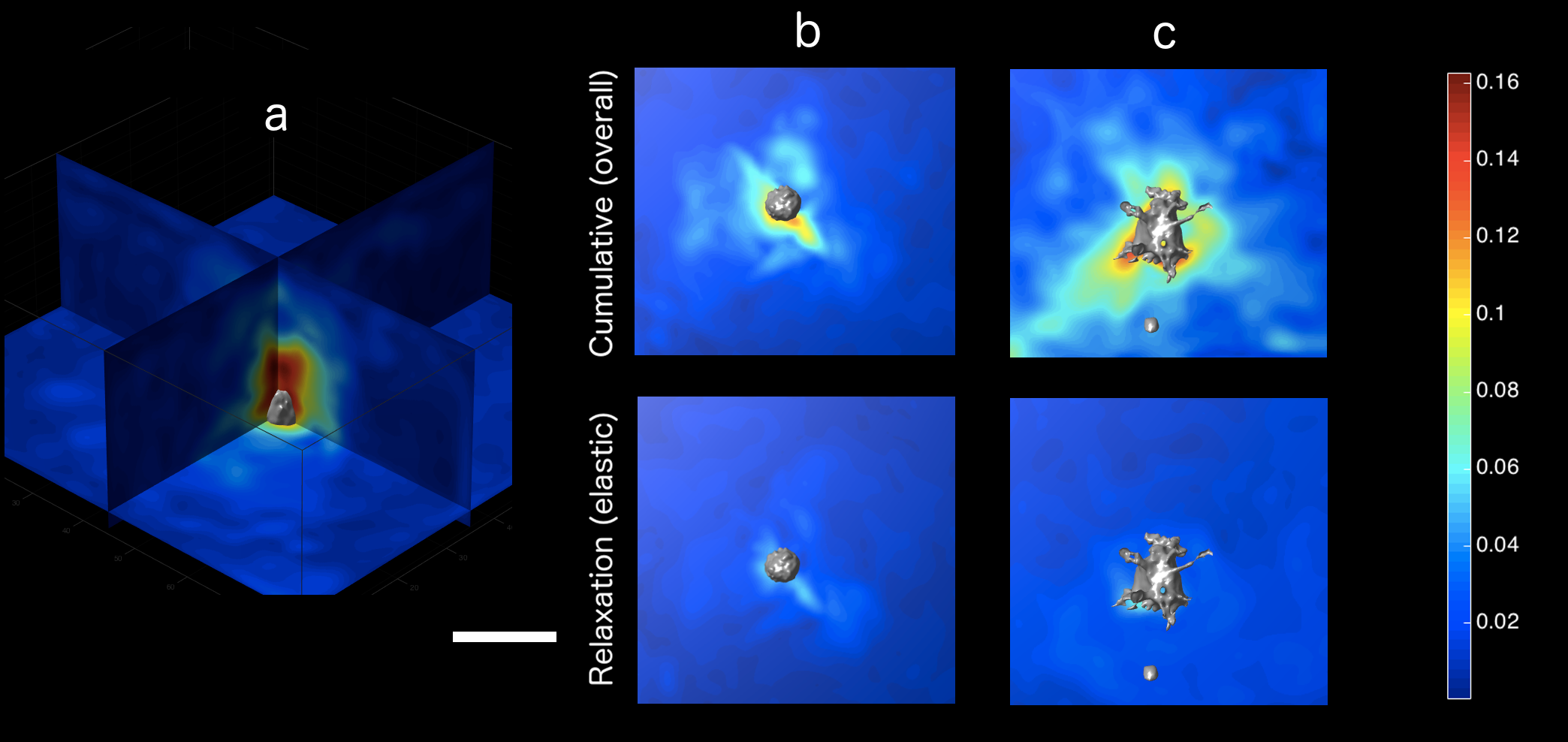

Supplement: S9 Fig — (a) Absolute maximum principal strain (AMPS) around an MDA-MB-231 cell in collagen in a 3D sliced view. (b) In-plane slice view of AMPS around an MDA-MB-231 in collagen ECM for (top) 4h experiment showing cumulative overall–plastic and elastic–deformation and (bottom) showing relaxation–elastic component–after 1h treatment with cell lysant. (c) In-plane slice view of cumulative and relaxation AMPS for HUVEC in fibrin ECM. (TIF) [file pcbi.1006684.s014.tif]

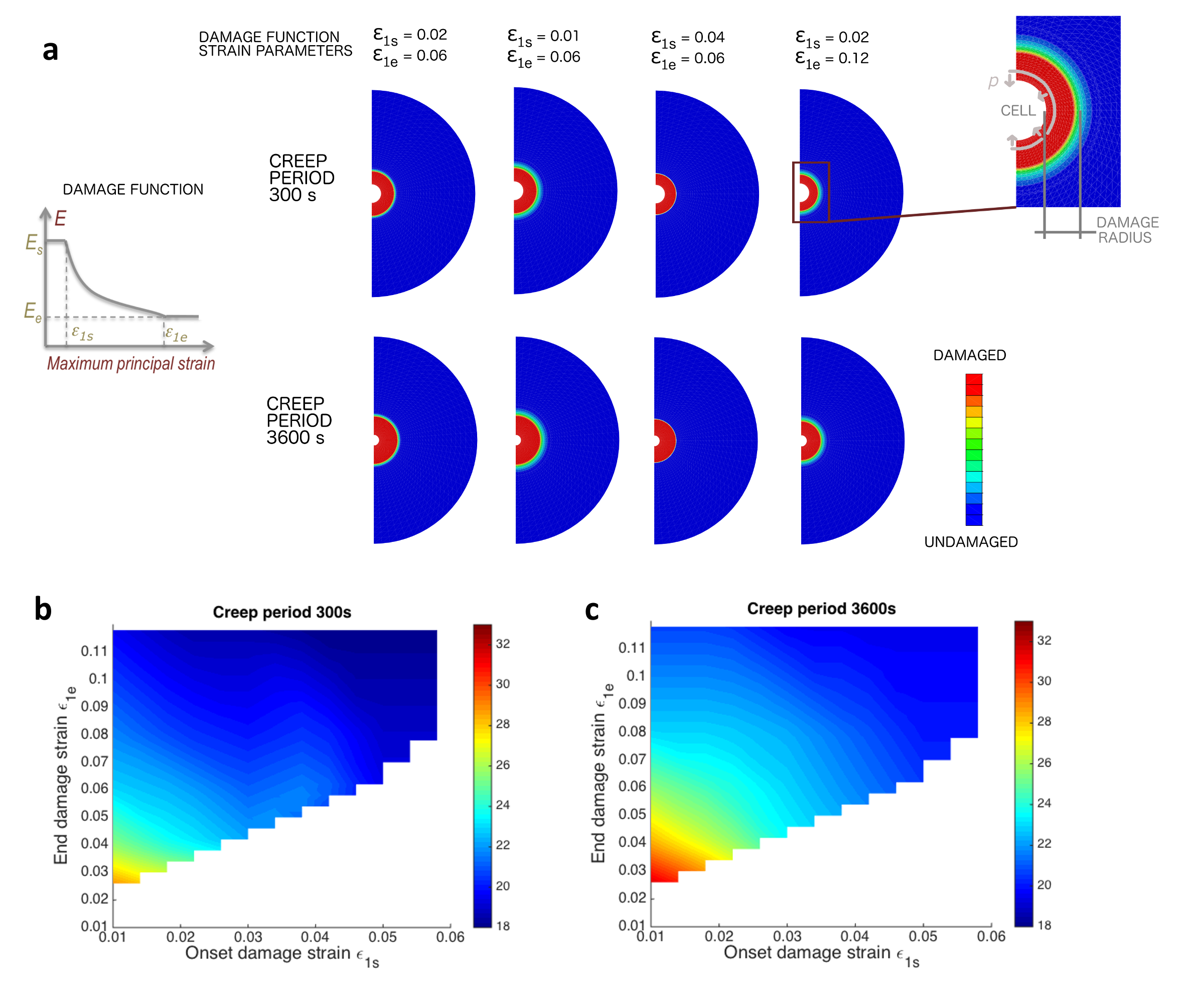

Supplement: S10 Fig — (a) Maximum principal strain parameters that control the damage model exponential decay (Fig 7B, reported also on the left) are varied. The baseline (ε1s = 0.02 or 2% and ε1e = 0.06 or 6% strains) and three additional variations are shown in the figure along with their effect on the spatial profile of the Young’s modulus (undamaged Young’s modulus Es is 1 kPa, and fully damaged Young’s modulus Ee is 0.2 kPa, see S2 Table). These plots are organized in two rows depending on whether the Young’s modulus damaging process is shown at the end of 300s creep (constant loading with p = 0.1 kPa), or 1 hour creep. On the right, a magnification shows how the damage radius is calculated, i.e. the radius up to regions with half-max damage. For clarity, the locations of the loading application and cell in the model are also indicated. (b) 2D heat map of damage radius as a function of the start, or onset damage strain ε1s and end strain ε1e at the end of 300s creep loading (p = 0.1 kPa) and (c) at the end of 1 hour creep. Note that the heat maps show reduced parameter space because the condition ε1s <ε1e must hold. (TIF) [file pcbi.1006684.s015.tif]

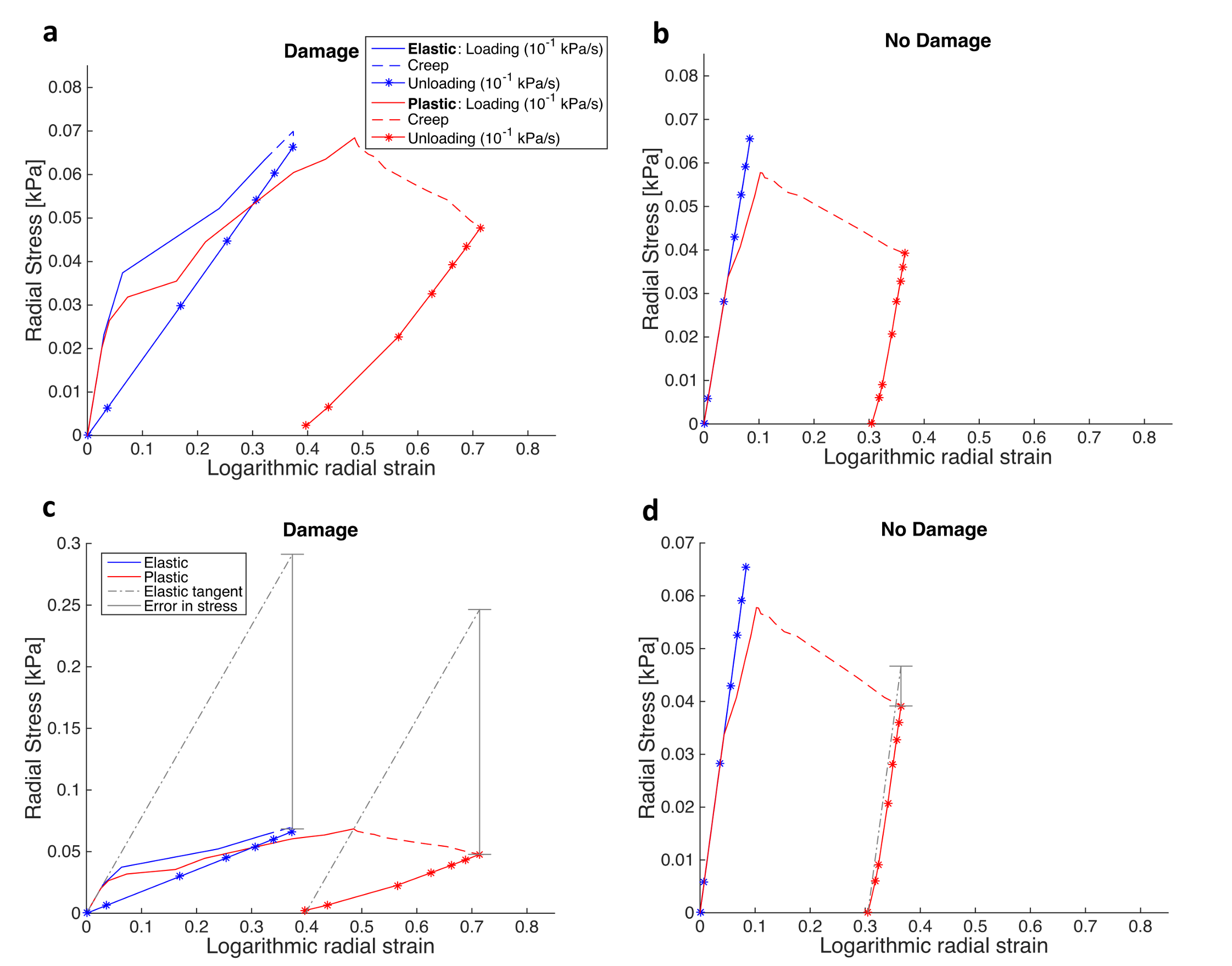

Supplement: S11 Fig — (a,b) Stress-strain response in viscoplastic (red) vs. purely elastic (blue) ECM continuum models. Different loading regimes are indicated along with loading rate (a) with damage and (b) without damage included in the constitutive material formulation. Note that in the viscoplastic models, plastic softening causes a drop in yield stress as plastic deformation increases during creep (creep period = 1500s, red dashed lines). (c,d) Local (at the load application region) errors in stress back-calculation based on comparing the viscoplastic and the purely elastic models of (a,b). All models have the same initial elastic stiffness: dashed grey lines represent the initial stiffness of undamaged Young’s modulus of ~1 kPa. (c) Comparison with damage in both elastic and viscoplastic formulations and (d) comparison without damage. Note that as expected, error is null with a purely elastic model with no damage (d, blue line) is calculated. The small error in d (red line) is due to a competition between softening (producing a negative slope during creep) and viscous effects during unloading. Note also that the stress axis limit is highly rescaled in (c) as compared to the same profiles in (a) to help visualize the magnitude of the error, indicated by the vertical solid line. (TIF) [file pcbi.1006684.s016.tif]

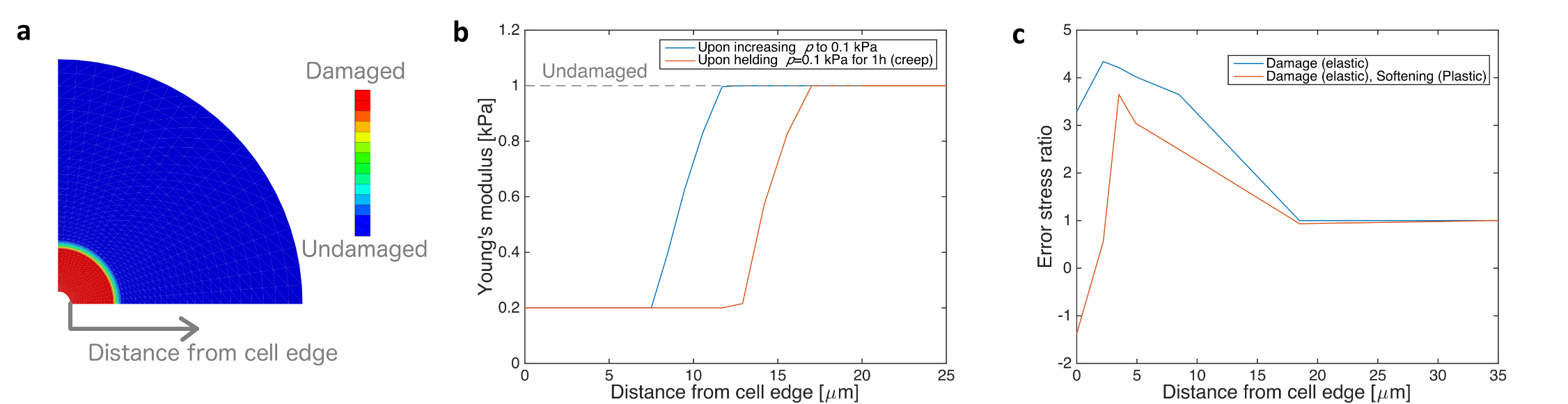

Supplement: S12 Fig — (a) Example of the spatial profile of the damaged Young’s modulus in the continuum model of the ECM. (b) Radial profile of Young’s modulus in two times of the load history: upon increasing the load p linearly from 0 to 0.1 kPa (blue), and at the end of 1 hour creep (red). (c) Spatial variation of the ratio between apparent stress–as if no ECM damage or plasticity was considered–and real stress. This ratio is estimated as in S11 Fig. (TIF) [file pcbi.1006684.s017.tif]

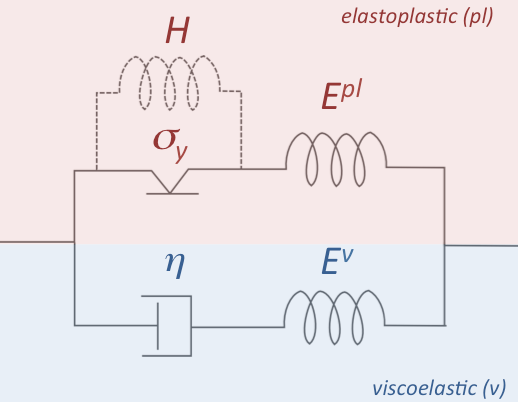

Supplement: S13 Fig — (TIF) [file pcbi.1006684.s018.tif]
