# Supplementary material for: Dynamic filopodial forces induce accumulation, damage, and plastic remodeling of 3D extracellular matrices
Source: PLoS Comput Biol. 2019 Apr 8;15(4):e1006684. doi: 10.1371/journal.pcbi.1006684 (PMC6472805; doi:10.1371/journal.pcbi.1006684)
Supplement: S1 Table — (DOCX) [file pcbi.1006684.s019.docx]

**S1 Table**: Parameters used for the discrete model simulations

| **Symbol** | **Definition** | **Value** |
| --- | --- | --- |
| $L_{f}$ | Length of cylindrical fiber segment | 800 [nm] |
| $D_{f}$ | Diameter of cylindrical fiber segment | 100 [nm] |
| $E_{f}$ | Young’s modulus of fiber | 125 [N m^-2^] |
| $L_{xlink}$ | Length of each crosslinking arm | 20 [nm] |
| $k_{u0}$ | Zero-force unbinding rate of crosslink | 3.4 x 10^-4^ [s^-1^] (1×) |
| $\lambda$ | Mechanical compliance of crosslink bond | 0.37 [nm] (1×) |
| $C_{f}$ | Fiber Concentration | 9024 segments per 20×20×20 μm^3^ |
| % Xlinks | Ratio Crosslink Concentration to $C_{f}$× 100 | 80 (1×) |
| $\eta$ | Viscosity of medium (water) | 0.86 × 10^-3^ [kg m^-1^ s^-1^ ] |
| $\Delta t$ | Time step of simulation | 2.379 × 10^-9^ [s] |
| $k_{B}T$ | Thermal energy | 4.142 × 10^-21^ [J] |
