# Supplementary material for: Dynamic filopodial forces induce accumulation, damage, and plastic remodeling of 3D extracellular matrices
Source: PLoS Comput Biol. 2019 Apr 8;15(4):e1006684. doi: 10.1371/journal.pcbi.1006684 (PMC6472805; doi:10.1371/journal.pcbi.1006684)
Supplement: S2 Table — (DOCX) [file pcbi.1006684.s020.docx]

**S2 Table**: Parameters used for the finite element continuum model simulations

| **Symbol** | **Definition** | **Value** |
| --- | --- | --- |
| $R_{c}$ | Radius of the cell | 10.0 [μm] |
| $R_{p}$ | Force application radius | 12.2 [μm] |
| $p$ | Stress applied | 0.1 [kPa] |
| $E$ | Young's modulus continuum material | 1.0 [kPa] |
| $\eta$ | Viscosity of the continuum | 10^2^ [kPa s] |
| *Damage model* | | |
| $E_{s}$ | Start damage modulus | 1.0 [kPa] |
| $\varepsilon_{1s}$ | Start damage strain | 0.02 |
| $E_{e}$ | End damage modulus | 0.2 [kPa] |
| $\varepsilon_{1e}$ | End damage strain | 0.06 |
| *Softening model* | | |
| $\sigma_{ys}$ | Start Yield stress | 0.03 [kPa] |
| $\varepsilon_{ps}$ | Start Plastic strain | 0 |
| $\sigma_{ye}$ | End Yield stress | 0.01 [kPa] |
| $\varepsilon_{pe}$ | End Plastic strain | 0.03 |
