# Supplementary material for: Dynamic filopodial forces induce accumulation, damage, and plastic remodeling of 3D extracellular matrices
Source: PLoS Comput Biol. 2019 Apr 8;15(4):e1006684. doi: 10.1371/journal.pcbi.1006684 (PMC6472805; doi:10.1371/journal.pcbi.1006684)
Supplement: S1 Note — (DOCX) [file pcbi.1006684.s021.docx]

**S1 Note:** Viscoplasticity theory and finite element calculations

**1. Continuum model for the viscoplastic behavior**

To model a continuum viscoplastic response, the material must include elastic, plastic, and viscous components. A one-dimensional schematic of the constitutive model is shown in Figure 7a in a simplified version and in S13 Fig in a more complete form, with the elastoplastic and the viscoelastic components in parallel. The hardening/softening element is also schematized in S13 Fig.

A linear isotropic response is specified for both elastic components. One can define the ratio $f$ of the elastic modulus of the elastic-viscous component ($E^{v}$) to the total elastic modulus ($E^{v}+E^{pl}$). The Poisson's ratios are the same in both components, and the properties specified for the elastic behavior are to be intended as instantaneous properties.

We modeled the viscoplastic behavior using the Norton-Hoff assumption. In this model, the total strain tensor ($\boldsymbol{\varepsilon}$) is the contribution of elastic ($\boldsymbol{\varepsilon}^{el}$), plastic ($\boldsymbol{\varepsilon}^{pl}$) and viscous($\boldsymbol{\varepsilon}^{v}$) strain tensors as follows:

$$\boldsymbol{\varepsilon}= \boldsymbol{\varepsilon}^{el}+ {(1-f)\boldsymbol{\varepsilon}}^{pl}+{f\boldsymbol{\varepsilon}}^{v} \left( 1 \right)$$

The viscoplastic coupling and creep behavior of a Norton-Hoff material can be understood in the one-dimensional idealization as:

$$\frac{d\varepsilon^{v}}{dt}=A\sigma_{V}^{n} \left( 2 \right)$$

where $\sigma_{V}$ is the stress in the viscous component and the rate parameters $A$ and $n$ represent the slope of the stress-strain curve of the viscous component and an exponential fit parameter, respectively. We have assumed a simple linear behavior with $n=1$ in Eq. 2 so that in the one-dimensional, perfect viscoplastic solid assumption, we have $A=\frac{1}{\eta}$ giving a relationship between the slope and the viscosity$\eta$ of the dashpot.

**2. Damage feature in a viscoplastic model**

In our implementation of the damage model, the Young’s modulus $E^{i}, i=v,pl$ is a function of the elastic maximum principal strain. Because in the simulated loading of a contracting cell, the maximum principal elastic strain is positive farther away from where the load is applied (Fig. 7c), the effect of damage translates to a degradation of the Young’s modulus as the material experiences tensile deformations. Close to the cell edge, local compression-related damage is present, but the characterization of this small region is out of the scope of the present study. Damage thus is meant to mainly simulate the breaking of crosslinks as the ECM fiber network is strained under tension, leading to degradation in the elastic properties. As shown in Figure 7b, for each elastic component $i$, four values are needed to fit an exponential drop for the Young’s modulus as a function of maximum tensile elastic strain, which characterize the start ($E_{s}^{i}, \varepsilon_{1, s}$) and end ($E_{e}^{i}, \varepsilon_{1, e}$) of the exponential damage law after which we assume that no further damage is possible. For simplicity we consider, in Eq. 1, $f=\frac{E^{v}}{E^{v}+E^{pl}}=0.5$, such that $E^{v}=E^{pl}=E$, providing a single Young’s modulus damage definition for all elastic components in the constitutive model.

**3. Softening in a viscoplastic model**

Softening is modeled through the definition of a negative slope in a standard plastic hardening behavior ­— represented by the parameter $H$. We have chosen a linear softening, *i.e.* a linear dependence between the yield stress $\sigma_{y}$ and the plastic equivalent strain $\varepsilon^{pl}=\int_{0}^{t} \sqrt{\frac{2}{3}\frac{d\varepsilon^{pl}}{dt}\cdot\frac{d\varepsilon^{pl}}{dt}}dt$. We have assumed a linear drop in yield stress (Fig. 7b) from an initial state ($\sigma_{y,s}, \varepsilon_{s}^{pl}$) to a end state ($\sigma_{y,e}, \varepsilon_{e}^{pl}$) above which no further drop in yield stress can occur.

**4. Finite element implementation**

The viscoplastic model described in Section **1** and the softening described in Section **3** are implemented in the finite element package ABAQUS (Dassault Systèmes, Providence RI) using the built-in two-layer viscoelasticity module. For the damage model, the drop in Young’s modulus as a function of maximum tensile strain is implemented using the *DEPENDENCIES keyword for the Young’s modulus material parameter, and the exponential function is coded in the customizable user subroutine USDFLD. The contracting cell is modeled using an axisymmetric mesh. Only half of the cell is shown in Fig. 7c highlighting the spherical symmetry of the problem. The mesh is composed of 1720 4-node axisymmetric elements (CAX4). As described in the main text, the load is applied as a distributed pressure over a surface (Fig. 7c). The load profile includes three steps. First, an initial load increases linearly from 0 to $p$ in 10 seconds. Next, the load is maintained at $p$ for a creep period of 3000 seconds (except when this parameter is studied for sensitivity, Fig. 7i). Finally, the load is removed and the simulation is continued for >10^4^ seconds to ensure plateauing. Unless otherwise indicated, the loading and unloading rates are kept at 10^-2^ kPa/s. All parameters used in the model are reported in Supplementary Table 2.

**Supplementary References**

1. ABAQUS Analysis User’s Manual, Section 11.2.11 Two-layer viscoplasticity
2. R. Temam, “A generalized Norton-Hoff model and the Prandtl-Reuss law of plasticity”, *Arch. Mech. Rational. Anal.*, Vol. 95, pp. 137-183, 1986
